# Deciphering linezolid-induced hematologic toxicity: Targeting TOP2A and TOP2B via its primary metabolite PNU142586

**DOI:** 10.1126/sciadv.adt5833

**Published:** 2025-05-28

**Authors:** Vo Thuy Anh Thu, Nguyen Quynh Nhu, Nguyen Thi Van Anh, So-An Lim, Hyeon-Jeong Seong, Jony Md Rasheduzzaman, Uijin Kim, Hyun-Soo Cho, Soedarsono Soedarsono, Jae-Gook Shin, Yong-Soon Cho

**Affiliations:** ^1^ Center for Personalized Precision Medicine of Tuberculosis, Inje University College of Medicine, Busan, Korea.; ^2^ Department of Pharmacology and PharmacoGenomics Research Center, Inje University College of Medicine, Busan, Korea.; ^3^ Department of Systems Biology, Yonsei University, Seoul 03722, Republic of Korea.; ^4^ Institute for Bio-medical Convergence Science and Technology, Yonsei University, Seoul 03722, Republic of Korea.; ^5^ Sub-pulmonology Department of Internal Medicine, Faculty of Medicine, Hang Tuah University, Surabaya, Indonesia.; ^6^ Dr. Soetomo Academic General Hospital, Surabaya, Indonesia.; ^7^ Department of Clinical Pharmacology, Inje University Busan Paik Hospital, Busan, Korea.

## Abstract

Linezolid, an oxazolidinone antibiotic, is widely used to treat multidrug-resistant tuberculosis and drug-resistant Gram-positive infections. However, prolonged use is associated with severe hematologic toxicity, the underlying mechanisms of which remain incompletely understood, particularly regarding the role of linezolid metabolites. Our clinical study indicates that elevated exposure to PNU142586, a primary metabolite of linezolid, is associated with an increased risk of linezolid-induced toxicity, even in the absence of renal impairment. To elucidate its mechanism, we identify DNA topoisomerase 2-α (TOP2A) and DNA topoisomerase 2-β (TOP2B) as primary targets of PNU142586 at molecular, cellular, and in vivo levels. PNU142586 disrupts replication and transcription by impeding DNA binding to TOP2A and TOP2B with a favorable conformation for cleavage and by inhibiting adenosine 5′-triphosphate hydrolysis, ultimately leading to antiproliferative and cytotoxic effects, including mitochondrial dysfunction. The present study thus provides mechanistic insight into linezolid-induced hematologic toxicity and offers a foundation for safer antibiotic development and improved clinical monitoring through biomarker identification.

## Teaser

PNU142586, the major linezolid metabolite, predominantly targets TOP2A and TOP2B, leading to linezolid-induced severe toxicity.

## INTRODUCTION

The antibacterial effect of linezolid (LZD), a synthetic oxazolidinone antibiotic, occurs via the selective inhibition of bacterial protein synthesis (*1*). It binds to the 23S ribosomal RNA within the 50S subunit of the bacterial ribosome, which disrupts the assembly of the functional 70S initiation complex, hindering the formation of bacterial protein chains (*1*, *2*). LZD exhibits bacteriostatic activity against Gram-positive bacteria, including multidrug-resistant strains such as methicillin-resistant *Staphylococcus aureus* and vancomycin-resistant enterococci (*3*, *4*). Its ability to impede protein synthesis also extends its efficacy to slow-growing bacterial species such as *Mycobacterium tuberculosis*, the causative agent of tuberculosis (*5*, *6*). LZD primarily exhibits bacteriostatic activity against replicating *M. tuberculosis*, while it also demonstrates sterilizing activity, which may inhibit the survival of nonreplicating bacteria (*7*–*11*). These properties make LZD particularly effective against rifampicin-resistant tuberculosis (RR-TB) and multidrug-resistant tuberculosis (MDR-TB), which represent a major challenge in global tuberculosis management (*12*, *13*). Furthermore, in combination therapy alongside other potent antituberculosis agents, LZD has shown promise in enhancing treatment outcomes for patients afflicted with RR-TB and MDR-TB (*14*, *15*).

While LZD has demonstrated its effectiveness in treating RR-TB and MDR-TB, its use has been linked to peripheral neuropathy or hematologic adverse reactions, including thrombocytopenia, anemia, and myelosuppression (*5*). Especially, the occurrence rate of LZD-induced thrombocytopenia varies from 0.3 to 32%, while anemia incidence ranges from 3.6 to 36% (*16*–*19*). Several risk factors have been identified, such as prolonged treatment exceeding 28 days, concurrent administration of other drugs with myelotoxic potential, renal dysfunction, prior bone marrow suppression, and exceeding an LZD trough plasma concentration of ~8 mg/liter during short-term use or 2 mg/liter during long-term use (*5*, *20*–*22*). In conjunction with these risk factors, LZD exhibits concentration-dependent toxicity, with studies indicating a strong correlation between higher plasma concentrations of LZD and the development of hematologic toxicity, including thrombocytopenia, anemia, and myelosuppression (*17*, *20*, *22*, *23*). This supports the proposal of therapeutic trough concentration thresholds to optimize LZD’s safety profile. It has also been shown that the discontinuation of LZD typically results in the reversal of its toxicity (*5*, *16*, *17*, *19*).

Although the exact mechanisms responsible for LZD-induced hematologic adverse events remain unclear, current evidence suggests that LZD-induced mitochondrial toxicity may be a key contributor, similar to the mechanism observed for chloramphenicol-induced myelosuppression (*4*, *5*, *17*, *24*). Given the close similarity of mammalian mitochondrial ribosomal components and their bacterial counterparts targeted by LZD, it is plausible that LZD inhibits mitochondrial protein synthesis within host cells, leading to mitochondrial toxicity (*24*–*26*). The inhibition of mitochondrial ribosomes appears to be positively correlated with LZD exposure and has been associated with both the time- and concentration-dependent suppression of cell proliferation (*25*–*27*). This inhibition also occurs via a reduction in the expression of mitochondrial cytochrome c-oxidase subunit I (MT-CO1) (*25*). These findings suggest a potential mechanistic link between LZD exposure, mitochondrial dysfunction, and hematologic adverse events.

LZD is metabolized primarily through hepatic or chemical oxidation of its morpholine ring, yielding two inactive open-ring carboxylic acid derivatives: aminoethoxy acetic acid (PNU142300) and hydroxyethyl glycine (PNU142586) (fig. S1) (*28*). LZD and its metabolites are primarily excreted in the urine, with ~30% of each dose excreted unchanged (*28*). Renal impairment is independently associated with a heightened risk of hematologic adverse events such as thrombocytopenia, likely due to the greater accumulation of PNU142300 and PNU142586 compared to the parent drug (*17*, *28*–*30*). Thus, despite the limited understanding of the precise mechanisms underlying the toxicity associated with these metabolites, emerging evidence such as this suggests that they are involved in LZD-induced hematologic adverse events, emphasizing the need for further investigation. In addition, it appears that mitochondrial toxicity from the parent drug alone may not fully account for these adverse events. Therefore, identifying the specific targets of LZD metabolites is important to understand the mode of action underlying LZD-induced hematologic adverse events and to develop second-generation oxazolidinone derivatives with an improved safety profile.

In line with this, in the present study, we sought to identify specific host targets affected by LZD metabolites and thus understand the mode of action underlying LZD-induced hematologic adverse events. Using reverse in silico screening, transcriptomic signature analysis, and molecular dynamics (MD) simulations, we explored potential targets for LZD metabolites, ultimately pinpointing DNA topoisomerase 2-α (TOP2A) and DNA topoisomerase 2-β (TOP2B) as the key biologically relevant targets. This focus was informed by clinical data showing that elevated exposure to PNU142586, a primary metabolite of LZD, is associated with an increased risk of LZD-induced toxicity, even in patients without renal impairment. These findings were further corroborated by a series of experimental assays, including in vitro enzyme assays, cell-based assays, *Xenopus* oocyte experiments, and in vivo zebrafish system analysis. We also elucidated the molecular mechanisms by which the metabolites inhibit TOP2A and TOP2B, providing valuable insights into the underlying processes contributing to LZD-induced hematologic adverse events.

## RESULTS

### The exposure of LZD metabolites, particularly PNU142586, was associated with LZD-induced toxicity in the clinical study

We first undertook a human pharmacokinetic (PK) study to investigate the association between LZD-induced toxicity and its metabolites. In a clinical cohort study, we measured the drug concentrations of LZD and its metabolites in plasma samples from patients without renal impairment who experienced LZD-induced toxicity or those who did not. Using a previously reported population PK model for LZD and its two major metabolites (*31*), we predicted their exposure represented by the area under the concentration-versus-time curve (AUC) for both groups.

Previous research has established a close relationship between renal impairment and LZD-induced toxicity, noting that patients with renal impairment exhibit higher concentrations of LZD and its metabolites (PNU142586 and PNU142300), with metabolite levels being particularly sensitive to renal function (*32*, *33*). Our study focused on patients with normal renal function (estimated glomerular filtration rate ≥60 ml/min per 1.73 m^2^) to investigate the relationship between LZD-induced toxicity and metabolite exposure, independent of renal function.

The study included patients with various manifestations of LZD toxicity, not limited to hematologic toxicity. This broader inclusion was due to the relatively small number of patients experiencing hematologic toxicity alone. Given the shared potential mechanisms for LZD-induced hematologic toxicity and other toxic effects such as peripheral neuropathy and lactic acidosis, exemplified by mitochondrial toxicity known to contribute to all those conditions, this approach was considered reasonable (*34*–*37*).

**Fig. 1.**
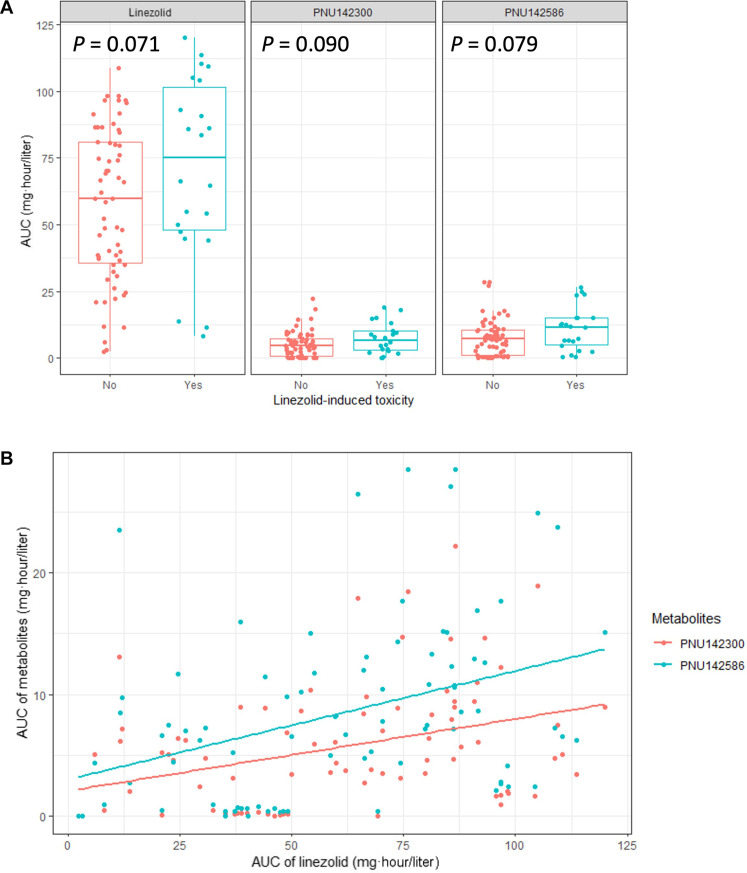
The exposure of LZD metabolites, particularly PNU142586, was associated with LZD-induced toxicity in the clinical study. (**A**) AUCs of LZD, PNU142300, and PNU142586 stratified by LZD-induced toxicity. (**B**) Linear regression between AUCs of LZD and metabolites. The linear correlation between AUCs of LZD and PNU142300 was 0.059 × LZD + 2.091 (*r*^2^ = 0.13). The linear correlation between AUCs of LZD and PNU142586 was 0.089 × LZD + 3.031 (*r*^2^ = 0.14).

The median (interquartile range) age, weight, and height of the patients were 41 years (26 to 52 years), 47.0 kg (41.0 to 54.3 kg), and 157.8 cm (153.0 to 164.0 cm), respectively. The AUCs of LZD and its two major metabolites are provided in Table 1. Compared with the patients who did not experience LZD-induced toxicity, those who did had median AUCs of LZD that were 1.25-fold higher, whereas median AUCs of PNU142586 and PNU142300 were 1.64-fold and 1.46-fold higher, respectively (Fig. 1A). However, because of the sample size of 84 patients (22 with toxicity versus 62 without toxicity), the results did not reach statistical significance but were marginally significant (*P* = 0.071 for LZD and *P* = 0.079 for PNU142586), indicating the need for a larger clinical study to clarify these findings.

**Table 1.** AUCs of LZD and its primary metabolites stratified by LZD-induced toxicity.

Variables	Total	LZD toxicity	
		No	Yes
No. of patients	84	62	22
AUC (mg·hour/liter)*			
LZD	63.4 (37.7–86.2)	59.9 (35.6–81.0)	75.0 (48.0–101.5)
PNU142300	4.8 (1.7–8.7)	4.6 (0.6–7.1)	6.7 (2.9–10.1)
PNU142586	7.2 (2.4–12.1)	7.1 (1.2–10.6)	11.6 (5.1–15.1)

*Data are presented as medians (interquartile range).

Our results indicated that patients who experienced LZD-induced toxicity had higher AUCs for both LZD and its primary metabolite compared to those without toxicity, even in the absence of renal impairment. Despite the limited sample size, the data revealed that the increase in exposure was more pronounced for the metabolites than for LZD itself. Notably, the increase in exposure was greater for PNU142586 than for PNU142300. This observation aligns with recent findings from experiments using megakaryocytic cell lines, which suggested that elevated levels of PNU142586 contribute to LZD-induced thrombocytopenia (*38*). Moreover, the correlation between AUCs of LZD and each metabolite was poor (*r*^2^ = 0.14 for PNU142586 and 0.13 for PNU142300) (Fig. 1B), suggesting that, regardless of renal function, the LZD metabolite, especially PNU142586, could play a critical role independent of LZD in the development of LZD-induced toxicity. However, it is important to acknowledge that our approach assumes a linear correlation between the parent drug and its metabolites. This assumption may oversimplify the dynamics of multimetabolite pathways, which could involve higher-order relationships and be influenced by external conditions. This limitation underscores the need for caution in interpreting these findings. Despite this limitation, our results necessitated further exploration into the molecular mechanisms underlying LZD-induced toxicity, leading us to perform in silico screening to identify potential targets of LZD metabolites.

### TOP2 was identified as the most relevant target of LZD metabolites using in silico screening

In silico inverse docking is a potent screening methodology that can be used to explore new target proteins for existing drugs and understand the molecular mechanisms underlying their clinical effects or potential toxicity while also minimizing the cost and time traditionally associated with experimental approaches (*39*). To identify potential host protein targets for LZD and its metabolites PNU142586 and PNU142300, we used two inverse docking platforms, SePreSA and ACID, as primary screening resources. Using SePreSA, a server housing an extensive array of structural models covering prominent targets associated with serious adverse drug reactions (*40*), we successfully identified six, three, and six protein targets for LZD, PNU142586, and PNU142300, respectively, based on the *Z* (<−1.2) and *Z*′ score (<−0.5) thresholds recommended by the server (*40*). Table 2 presents the identified proteins and their *Z* and *Z*′ scores. Monoamine oxidase (MAO) B emerged as a noteworthy target for LZD. Given the documented nonselective MAO-inhibitory activity associated with LZD, this finding supports the relevance of the docking process (*41*). Four proteins (nicotinamide *N*-methyltransferase, histamine *N*-methyltransferase, glutathione reductase, and MAO B) for LZD, one protein (hypoxanthine-guanine phosphoribosyltransferase) for PNU142586, and one protein [adenosine triphosphatase (ATPase) domain of TOP2A] for PNU142300 displayed markedly higher scores compared to other identified targets, with *Z* scores of <−2.0 or *Z*′ scores of <−0.5.

**Table 2.** Potential human protein targets of LZD and LZD metabolites PNU142586 and PNU142300 predicted using SePreSA.

Compound	PDB ID	Protein name	*Z* score	*Z*′ score
LZD	6PVS	Nicotinamide *N*-methyltransferase	−2.18	−1.28
	1GRE	Glutathione reductase	−2.10	−1.33
	2AOV	Histamine *N*-methyltransferase	−1.23	−1.11
	1WOK	Poly(ADP-ribose) polymerase (PARP)	−1.33	−0.86
	1GOS	Monoamine oxidase B	−2.29	−0.54
	2J4E	Inosine triphosphate pyrophosphatase	−1.25	−0.70
PNU142586	1BZY	Hypoxanthine-guanine phosphoribosyltransferase	−2.33	−1.43
	1I10	Lactate dehydrogenase	−1.37	−0.53
	3JXU	Heat shock protein 70	−1.39	−0.51
PNU142300	1ZXM	DNA topoisomerase 2-α	−3.00	−1.52
	2J4E	Inosine triphosphate pyrophosphatase	−1.42	−0.93
	3JXU	Heat shock protein 70	−1.53	−0.74
	1BZY	Hypoxanthine-guanine phosphoribosyltransferase	−1.49	−0.66
	1I10	Lactate dehydrogenase	−1.44	−0.64
	1GRE	Glutathione reductase	−1.75	−0.62

We then determined whether the targets identified using SePreSA were also found using ACID, a broader inverse docking server covering more than 800 approved drug targets (*42*). Of the top-scoring nonbacterial protein targets identified using ACID for each compound, three targets (nicotinamide *N*-methyltransferase, MAO B, and TOP2A ATPase domain) were also identified using SePreSA, all falling within the category of higher-scoring targets (table S1). However, because histamine *N*-methyltransferase and heat shock protein 70, which were targets identified by SePreSA, were absent from the ACID database, these two targets, along with the three targets identified by both docking platforms, were used for further investigation.

Of the five potential targets identified for LZD and its metabolites, TOP2A has previously been associated with hematologic toxicity, particularly myelosuppression induced by its inhibitor (*43*, *44*). However, the other targets lack current evidence linking them to hematologic adverse events. Nicotinamide *N*-methyltransferase, which is primarily expressed in the liver, participates in xenobiotic metabolism with a broad substrate spectrum; thus, it has a low likelihood of causing hematologic toxicity through inhibition (*45*). MAO B catalyzes the oxidative deamination of both biogenic and xenobiotic amines, primarily affecting the catabolism of neuroactive and vasoactive amines in the central nervous system and peripheral tissues. This enzyme plays a crucial role in conditions such as Alzheimer’s disease, Parkinson’s disease, and stress-induced cardiac damage rather than hematologic events (*46*–*48*). Histamine *N*-methyltransferase regulates one of histamine’s major metabolic pathways, affecting various physiological functions, but it is unlikely to be associated with hematologic adverse events (*49*). Heat shock protein 70, a conserved molecular chaperone, plays a crucial role in protein folding and is abundantly expressed in malignancies, making it a promising therapeutic target in cancer treatment without causing serious toxicity (*50*). Notably, TOP2A exhibited markedly higher docking scores for the metabolites in both SePreSA and ACID analyses, underscoring its potential as a key target for LZD metabolites. Moreover, given the high sequence similarity (90%) in the catalytic domain between TOP2A and its paralog TOP2B, which includes regions capable of drug binding, TOP2B may also serve as a potential target.

### Identifying TOP2 as a potential target of LZD-induced toxicities through transcriptomic signature analysis

Following the identification of potential target proteins using in silico inverse docking, where TOP2 was selected as a target based on biological relevance, additional insights were sought through a complementary approach. To further explore potential targets, we used the SigCom LINCS tool (*51*), a web-based search engine that processes and analyzes more than 1.5 million gene expression signatures from databases like the Library of Integrated Network-Based Cellular Signatures (LINCS), the Genotype-Tissue Expression (GTEx), and Gene Expression Omnibus (GEO). This tool enabled us to conduct a large-scale chemically induced transcriptome analysis, focusing on the transcriptomic signatures induced by LZD at clinically relevant concentrations (10 and 3.3 μM) in the human blood cell line THP-1. Although direct transcriptomic data from THP-1 cells treated with LZD metabolites are unavailable, it is noteworthy that THP-1 cells constitutively express CYP2J2 and CYP1B1 (*52*, *53*), the major enzymes involved in LZD metabolism (*54*). In addition, nonenzymatic metabolism is also possible within these cells. This suggests that the transcriptomic profile obtained from LZD-treated THP-1 cells likely encompasses the effects of its metabolites.

The signature similarity search revealed that, among the top 10 drugs whose transcriptomic profiles closely mimicked those of LZD at both concentrations, a statistically significant commonality (*P* < 5 × 10^−8^) was identified with the fluoroquinolone class, specifically moxifloxacin at 10 μM and clinafloxacin at 3.3 μM (table S2). While fluoroquinolones are traditionally recognized for targeting prokaryotic type II topoisomerases, they are also known to inhibit eukaryotic TOP2 (*55*). This observation is consistent with the targets identified through our in silico inverse docking approach, further validating the relevance of eukaryotic TOP2 as a potential target in the context of LZD-induced hematologic toxicities. This convergence of evidence from both protein-based target identification and cell-based signature analysis strengthens the validity of the selected targets and underscores the utility of a multifaceted approach in drug-target interaction studies. Consequently, on the basis of these findings from in silico inverse docking and chemically induced transcriptome analysis, our focus shifted to TOP2A and its paralog TOP2B as the most promising targets for LZD metabolites.

### Targeted docking and MD simulation confirm potential interaction between LZD metabolites and TOP2

**Fig. 2.**
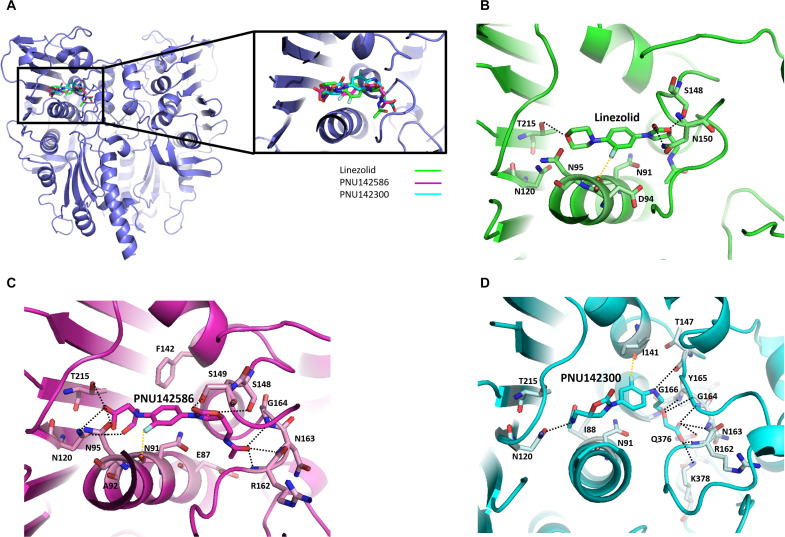
Targeted docking confirms the potential interaction between LZD metabolites and TOP2. (**A**) Comparison of LZD (green) and LZD metabolites PNU142586 (magenta) and PNU142300 (cyan) docked to the TOP2A ATPase domain (PDB ID: 1zxm) with a magnified view of the binding between the ligands and protein at the active site. The protein is depicted as a blue cartoon model. Analysis of interactions between the binding site of the TOP2A ATPase domain and LZD (**B**), PNU142596 (**C**), and PNU142300 (**D**). All residues that interact with the corresponding compound are presented as sticks. Hydrogen bonds are shown by black dotted lines, and halogen bonds are represented by yellow dotted lines. Hydrogen atoms are omitted for clarity.

In our investigation, targeted molecular docking was used to examine the interaction between TOP2A and LZD and its metabolites. Because detailed interactions between ligands and target proteins cannot easily be determined using inverse docking servers, we conducted a comprehensive analysis of the docking interactions of LZD and its metabolites within the binding pocket of the TOP2A ATPase domain using AutoDock Vina. The resulting overall binding modes are illustrated in Fig. 2A. Within the binding site, LZD formed hydrogen bonds with four amino acids (Asn^91^, Ser^148^, Asn^150^, and Thr^215^) and exhibited hydrophobic interactions with Asn^91^, Asp^94^, Asn^95^, and Asn^120^, including π stacking and halogen bonding with Asn^91^ (Fig. 2B). PNU142586 formed hydrogen bonds with eight amino acids (Asn^95^, Asn^120^, Ser^148^, Ser^149^, Arg^162^, Asn^163^, Gly^164^, and Thr^215^) and exhibited hydrophobic interactions with Glu^87^, Asn^91^, Ala^92^, Asn^95^, Phe^141^, and Ser^148^. In addition, a halogen bond with Asn^91^ was identified in the complex of the TOP2A ATPase domain and PNU142586 (Fig. 2C).

PNU142300 formed hydrogen bonds with eight amino acids (Asn^120^, Thr^147^, Arg^162^, Gly^164^, Tyr^165^, Gly^166^, Gln^376^, and Lys^378^) and exhibited hydrophobic interactions with Ile^88^, Asn^91^, Iie^141^, Asn^163^, Gly^164^, Thr^215^, and Lys^378^. Moreover, it formed a halogen bond with Ile^141^ (Fig. 2D). Docking results revealed that both metabolites of LZD, PNU142586 and PNU142300, were capable of forming a higher number of hydrogen bonds and an additional halogen bond with the TOP2A ATPase domain compared to LZD, with higher docking scores observed. While the TOP2A ATPase domain was initially identified as the highest-scoring target for PNU142300 in inverse docking screening, the forward-targeted approach revealed that PNU142586 also demonstrated robust interactions with the TOP2A ATPase domain. In addition, the docked orientation of the metabolites differed because of the accessibility of their respective moieties, resulting from the oxidation of the morpholine ring of LZD, to the deep binding pocket characterized by smaller and more rigid spaces. Notably, the size of the hydroxyethyl glycine moiety of PNU142586 was considerably larger than that of the aminoethoxyacetic acid moiety of PNU142300, potentially rendering it less favorable for placement within the binding pocket (fig. S1).

To assess the overall stability of the complex formed between the TOP2A ATPase domain and LZD and its metabolites, MD simulations were conducted. Analysis of the backbone root mean square deviation (RMSD) revealed that the TOP2A ATPase domain in all systems reached convergence within the extended simulation period, with distinct stability trends observed across the systems (fig. S2). Among these, the TOP2A ATPase domain-PNU142586 complex exhibited the lowest RMSD values for the majority of the simulation time after the initial equilibration period despite some fluctuations. This indicates that PNU142586 was the most stable interaction partner for the TOP2A ATPase domain. The combination of docking and MD simulation results collectively thus suggests that the TOP2A ATPase domain-PNU142586 complex is the most stable configuration, highlighting the potential of PNU142586 as the optimal interaction partner for the TOP2A ATPase domain.

### The LZD metabolite PNU142586 is an inhibitor of TOP2A and TOP2B

**Fig. 3.**
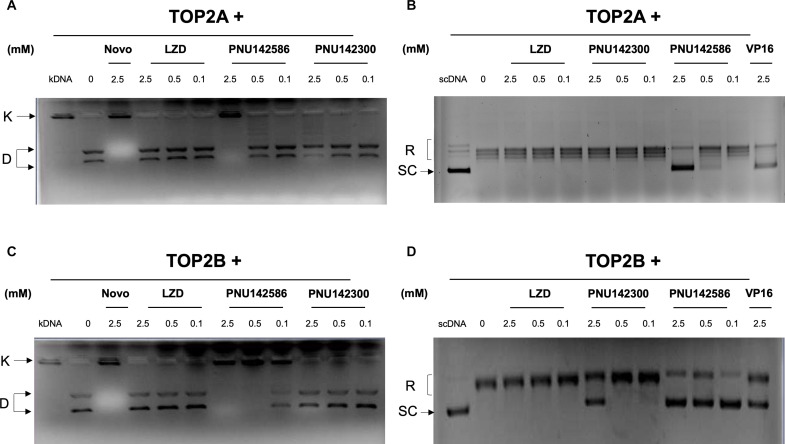
The LZD metabolite PNU142586 is an inhibitor of TOP2A and TOP2B. The kNDA decatenation activity of TOP2A (**A**) and TOP2B (**C**) was measured in the presence of LZD or its metabolites PNU142586 and PNU142300 (in mM). Novobiocin (Novo) was used as the positive control. The scDNA relaxation activity of TOP2A (**B**) and TOP2B (**D**) was measured in the presence of LZD or its metabolites PNU142586 and PNU142300 (in mM). Etoposide (VP16) was used as the positive control. K, catenated kDNA; D, decatenated kDNA; SC, supercoiled forms of the plasmid; R, relaxed forms of the plasmid.

To assess the potential of LZD and its metabolites as inhibitors of TOP2, kinetoplast DNA (kDNA) decatenation assays, which are the most specific assay available for TOP2, were conducted. PNU142586 demonstrated dose-dependent inhibition of TOP2A-mediated kDNA decatenation, with a median inhibitory concentration (IC_50_) of ~150 μM (55.40 mg/liter), which was comparable to the positive control novobiocin, a TOP2 inhibitor (Fig. 3A and fig. S3A). In contrast, LZD and PNU142300 did not exhibit any inhibitory activity in their assays (Fig. 3A). To further quantify the inhibitory effects on TOP2A, in vitro supercoiled DNA (scDNA) relaxation assays for TOP2 activity were conducted. PNU142586 exhibited a dose-dependent inhibition of TOP2A activity, inducing a shift from supercoiled to relaxed forms of plasmid DNA, with an IC_50_ of ~1.425 mM (Fig. 3B and fig. S3B). PNU142300 displayed limited inhibitory activity in terms of TOP2A-mediated relaxation, with a reduction of less than 50% at the highest tested concentration of 2.5 mM. In contrast, LZD did not exhibit any inhibitory activity (Fig. 3B). Similar trends were observed for the inhibition of TOP2B activity by LZD and its metabolites (Fig. 3, C and D). PNU142586 inhibited TOP2B, with IC_50_ values of ~150 μM (55.40 mg/liter) and 1.875 mM in the decatenation and relaxation assays, respectively (fig. S3, C and D). PNU142586 exhibited inhibition of both TOP2A and TOP2B but displayed a preference for inhibiting decatenation over relaxation for both TOP2A and TOP2B.

### PNU142586 is a catalytic inhibitor of TOP2

**Fig. 4.**
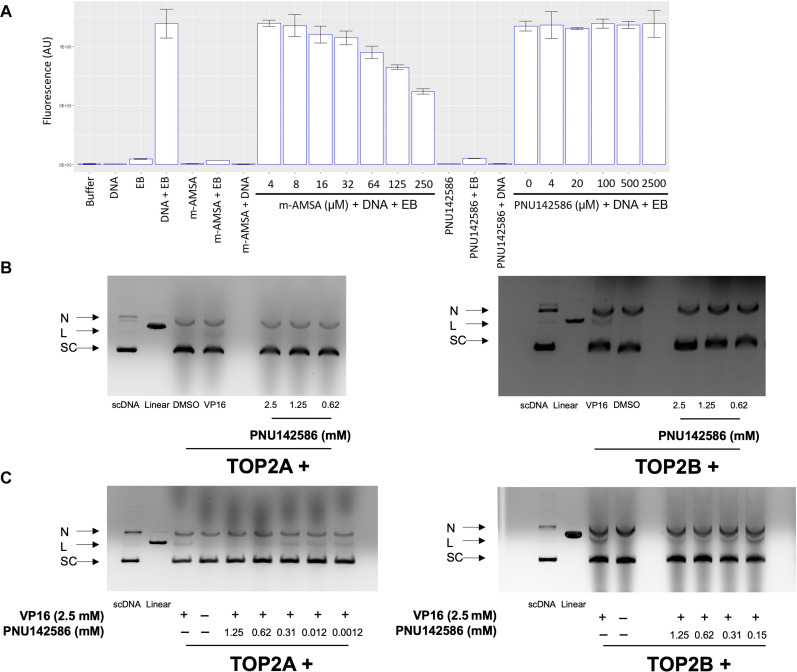
PNU142586 is a catalytic inhibitor of TOP2. (**A**) In ethidium bromide (EB) displacement assays, DNA (salmon sperm DNA, 100 μM) was mixed with or without ethidium bromide (2 μg/ml) together with the indicated chemicals. Reactions were subjected to fluorescence emissions of 590 nm and excitation of 535 nm. AU, arbitrary units. (**B**) DNA cleavage assays were conducted using human TOP2A and TOP2B incubated with etoposide (VP16, 2.5 mM), DMSO, or PNU142586 (in mM) in the presence of supercoiled plasmid DNA along with ATP. (**C**) DNA cleavage assays were conducted using human TOP2A and TOP2B incubated with the supercoiled plasmid DNA in the presence of VP16 and PNU142586 (in mM) in a reaction buffer with ATP. Reaction products from (B) and (C) were separated by electrophoresis of agarose gel containing ethidium bromide. N, nicked DNA; L, linear DNA; SC, scDNA; Linear, linear marker.

To determine the specific mechanism by which PNU142586 influences the catalytic process of TOP2, we conducted a series of complementary experimental assays. Initially, ethidium bromide displacement assays were conducted to assess the potential interaction of PNU142586 with DNA through the formation of intercalation complexes, which could potentially inhibit TOP2 activity. When bound to DNA, ethidium bromide exhibits markedly stronger fluorescence emissions compared to its free form. Consequently, the displacement of ethidium bromide from DNA can be monitored by the consequent decrease in fluorescence intensity. Our results demonstrated that increasing doses of m-AMSA (amsacrine), a reference intercalator, effectively reduced the fluorescence induced by the ethidium bromide/DNA complex, whereas PNU142586 did not induce this displacement (Fig. 4A). These findings indicate that PNU142586 does not intercalate with DNA and support the possibility that PNU142586 binds to TOP2 to inhibit its enzyme activity.

To ascertain whether PNU142586 acts as a catalytic TOP2 inhibitor, we conducted TOP2-mediated DNA cleavage assays. In these assays, both TOP2A and TOP2B were incubated with etoposide or PNU142586 in the presence of supercoiled plasmid DNA and adenosine 5′-triphosphate (ATP). The reaction products were separated using electrophoresis with agarose gel containing ethidium bromide. Unlike etoposide, PNU142586 did not generate detectable linear bands on the gel for either TOP2A or TOP2B, even at concentrations that completely inhibited TOP2 activity. This observation suggests that the mechanism of action for PNU142586 with TOP2 differs from that exhibited by TOP2 poisons such as etoposide, which stabilize the covalent TOP2-DNA cleavage complex (Fig. 4B).

Subsequently, DNA cleavage assays were conducted in the presence of etoposide to determine whether PNU142586 prevents the formation of the covalent TOP2-DNA cleavage complex or stabilizes the noncovalent TOP2-DNA complex following the formation of the covalent TOP2-DNA cleavage complex in the presence of a TOP2 poison. The addition of increasing concentrations of PNU142586 to reactions containing etoposide inhibited etoposide-induced DNA cleavage (Fig. 4C). This indicates that PNU142586 exerts its inhibitory effects by interfering with the formation of the covalent TOP2-DNA cleavage complex, thereby establishing its role as a catalytic inhibitor of both TOP2A and TOP2B.

### PNU142586 directly binds to TOP2

**Fig. 5.**
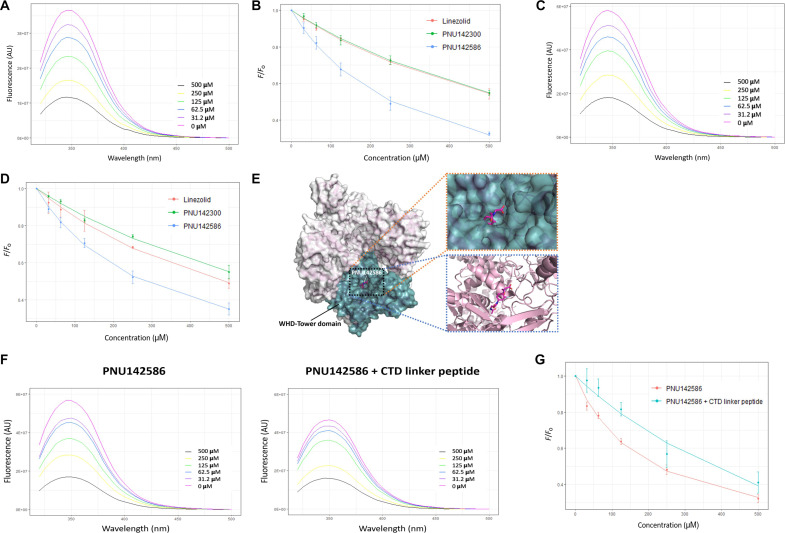
PNU142586 directly binds to TOP2. Trp fluorescence spectra of the TOP2A DNA binding domain (**A**) and ATPase domain (**C**) were recorded at increasing concentrations of PNU142586. Insets show the titrated concentrations. Fluorescence titrations with LZD, PNU142300, and PNU142586 at a single wavelength were normalized by dividing the measured fluorescence (*F*) by the fluorescence in the absence of the compound (*F*_o_) and plotted to assess binding affinities in the DNA binding domain (**B**) and ATPase domain (**D**). Solid lines represent fits to a site-specific binding model. (**E**) Surface representation of the TOP2A DNA binding domain (PDB ID: 5gwk) docked with PNU142586 in the putative drug-binding pocket is shown (green box). Magnified views highlight the pocket using surface (orange box) and cartoon (blue box) representations. (**F**) Trp fluorescence spectra of the DNA binding domain were measured with increasing concentrations of PNU142586 in the absence (left) or presence (right) of a CTD linker peptide. Insets show the titrated concentrations. (**G**) Fluorescence titrations of PNU142586, with and without the CTD linker peptide, were normalized to *F*_o_ and plotted to determine binding affinities. Solid lines indicate fits to a site-specific binding model.

To investigate the impact of PNU142586 on the catalytic process for TOP2, we conducted tryptophan (Trp) fluorescence quenching (TFQ) assays to monitor its direct binding to the DNA binding or ATPase domains of TOP2A, to which catalytic inhibitors are known to bind. Initially, we titrated the purified recombinant TOP2A DNA binding domain with PNU142586 and monitored fluorescence emissions in the range of 300 to 500 nm (excitation at 295 nm). The maximum Trp fluorescence of the TOP2A DNA binding domain occurred at 350 nm, and titration with PNU142586 resulted in a ligand concentration–dependent red shift in the fluorescence emission maxima, accompanied by a decrease in fluorescence intensity (Fig. 5A and fig. S4, A and B). The changes in Trp fluorescence as a function of the ligand concentration were plotted, and the binding affinity *K*_D_ was calculated to be 310.6 ± 89.2 μM (114.72 ± 32.95 mg/liter) (Fig. 5B). To further support the biological relevance of PNU142586 binding to the DNA binding domain of TOP2 observed in our TFQ experiments, we performed cryo–electron microscopy (cryo-EM) analysis of the recombinant DNA binding domain (table S3). The cryo-EM data confirmed that the DNA binding domain adopts a dimeric configuration, consistent with the structural arrangement of the full-length TOP2 homodimer in cells (fig. S10). This structural evidence reinforces the validity of the *K*_D_ values obtained from the TFQ assays, as the experiments were conducted using the biologically relevant dimeric form of the DNA binding domain.

Subsequently, we used similar TFQ assays to characterize the interaction of PNU142586 with the TOP2A ATPase domain. The maximum Trp fluorescence emission peak of the ATPase domain also occurred at 350 nm with excitation at 295 nm (Fig. 5C and fig. S4, C and D). Titration with increasing concentrations of PNU142586 resulted in an unchanged maximum emission wavelength (Fig. 5C). However, the intensity of the Trp fluorescence signal was quenched in a direct and dose-dependent manner with increasing amounts of the ligand, suggesting the direct binding of PNU142586 to the TOP2 ATPase domain, as predicted by our in silico docking analysis. The *K*_D_ value for PNU142586 binding to the ATPase domain was determined to be 300.8 ± 31.3 μM (111.10 ± 11.56 mg/liter) (Fig. 5D). PNU142586 thus exhibited a similar binding affinity with both the DNA binding and ATPase domains of TOP2A. Given the high sequence identity between the DNA binding and ATPase domains of TOP2A and TOP2B, it is possible that PNU142586 may also bind to the corresponding domains of TOP2B with similar affinity. Hence, our TFQ assay findings indicate that PNU142586 exerts its inhibitory effects on TOP2 through direct binding to both the DNA binding and ATPase domains, providing insights into its mode of action as a catalytic inhibitor of TOP2.

We also used in silico docking to investigate the potential binding of PNU142586 to the DNA binding domain of TOP2A. While few studies have been reported on potential drug-binding pockets in the DNA binding domain that substantially affect the interaction of TOP2A with DNA substrates, recent research by Matias-Barrios *et al.* (*56*) suggested a promising drug-binding pocket in the WHD-Tower domain (residues 721 to 1013) of this domain (fig. S5A), surrounded by specific amino acids that are highly conserved between TOP2A and TOP2B (*56*). In our investigation, PNU142586 formed a network of hydrogen bonds and hydrophobic interactions with key amino acids in this pocket, indicating its potential to disrupt TOP2-DNA interactions and inhibit TOP2 activity. The putative binding modes are presented in Fig. 5E and fig. S5B. Steric hindrances were also revealed between PNU142586 and the DNA substrate, with its hydroxyethyl glycine moiety overlapping with the position of the DNA backbone. In contrast, both LZD and PNU142300 exhibited relatively weak steric hindrance of the DNA substrates or did not overlap with them (fig. S5C), providing further insight into how PNU142586 modulates TOP2-DNA interactions.

To further evaluate the stability and molecular recognition of PNU142586 in the DNA binding domain of TOP2A, MD simulations were conducted for 200 ns, along with comparative simulations for LZD and PNU142300. The RMSD of the backbone was calculated to assess the stability of the complexes (fig. S5D). The simulation results revealed that the TOP2A DNA binding domain-PNU142586 complex exhibited the lowest RMSD values among all systems during the initial 100 ns, indicating significant stability within the binding pocket. At ~125 ns, the RMSD for the PNU142586 complex sharply increased, stabilizing at a new plateau for the remainder of the simulation. This behavior was unique to the PNU142586 system, as the RMSD values for the LZD, PNU142300, and Apo systems remained relatively stable or fluctuated within a narrower range. To investigate the structural basis of this RMSD shift, conformations at 0, 100, 125, and 200 ns were extracted for analysis (fig. S5E). The structures at 0 and 100 ns were highly similar, indicating a stable binding mode for PNU142586 within the DNA binding pocket during the initial phase of the simulation. At 125 ns, a conformational change in the DNA binding domain was observed, which correlated with the RMSD increase. Despite this change, PNU142586 remained securely bound within the pocket, ruling out reorientation or loosening of the ligand as the cause for the RMSD shift. By 200 ns, the structure had stabilized into a new conformation similar to the one observed at 125 ns (fig. S5F). This conformational change, which was not observed for LZD or PNU142300, suggests that PNU142586 induces a unique structural rearrangement in the DNA binding domain. Combined with its steric hindrance effects between PNU142586 and DNA in the initial status, this conformational change may play a critical role in modulating TOP2-DNA interactions, providing a potential explanation for the mechanism by which PNU142586 inhibits TOP2 activity.

In addition to the potential drug-binding pocket, an allosteric regulation site is present in the WHD-Tower domain of the DNA binding domain, as determined using cryo-EM structural analysis of TOP2A (*57*). The allosteric regulation site can be occupied by the CTD linker (residues 1191 to 1217), which flanks the DNA binding domain of TOP2A, stimulating its catalytic activity through structural changes. Intriguingly, the allosteric regulation site is in close proximity to the potential drug-binding pocket (fig. S5G), suggesting that the binding of the CTD linker to the allosteric regulation site could negatively affect the binding of PNU142586 to the drug-binding pocket.

To assess the influence of the allosteric regulation site on the binding of PNU142586 to the DNA binding domain, we conducted competition TFQ assays with the CTD linker peptide (residues 1193 to 1217) to observe changes in PNU142586’s binding affinity to the DNA binding domain in the absence and presence of this peptide. The maximum Trp fluorescence intensity of the TOP2A DNA binding domain titrated with PNU142586 in the presence of the CTD linker peptide decreased across all titrated PNU142586 concentrations compared with the absence of the peptide, and the binding affinity of PNU142586 decreased approximately fivefold (the mean *K*_D_ values without and with the CTD linker peptide were 190 and 1062 μM, respectively) (Fig. 5, F and G). These results indicate that the binding of the CTD linker to the allosteric regulation site in the WHD-Tower domain of the DNA binding domain can suppress the binding of PNU142586 to the DNA binding domain.

On the basis of the structural comparison between the full-length TOP2A (including the CTD linker) and the DNA binding domain only, the binding of the CTD linker to the allosteric regulation site appears to cause a local structural change in the WHD-Tower domain, thus acting as a positive regulator rather than inducing a global change in the DNA binding domain. Considering this, our results derived from the use of the CTD linker peptide provide indirect evidence for the binding of PNU142586 to the proposed drug-binding pocket in the WHD-Tower domain of the DNA binding domain.

### PNU142586 exhibits a dual molecular mode of action in inhibiting TOP2

On the basis of its ability to directly bind to both the DNA binding and ATPase domains of TOP2A, we investigated how these interactions potentially impede the formation of the covalent TOP2-DNA cleavage complex. In particular, we tested whether PNU142586 inhibits TOP2-mediated ATP hydrolysis or interferes with the binding of TOP2 to DNA, both of which are important steps in the catalytic process for TOP2. Initially, we evaluated the effect of PNU142586 on TOP2B-mediated ATP hydrolysis using purified full-length TOP2B in decatenation assays. In TOP2B-mediated kDNA decatenation assays at different ATP concentrations, the inhibitory effect of PNU142586 was slightly weakened at the highest tested concentration of ATP, which was indicative of relatively ATP-independent inhibition (fig. S6A). Considering the stronger inhibitory potency of PNU142586 in the decatenation and relaxation of TOP2, these findings suggest that, while PNU142586 binds to the ATPase domain and exhibits some inhibition of ATP hydrolysis via TOP2B, its primary mechanism of action likely involves binding to the DNA binding domain rather than inhibiting ATP hydrolysis.

To further explore the interaction between PNU142586 and TOP2, we investigated its influence on the noncovalent interaction between TOP2A and DNA using electrophoretic mobility shift assays (EMSAs) with the purified DNA binding domain of TOP2A. Even at high concentrations, PNU142586 did not abolish the shift in the biotin-labeled double-stranded DNA (dsDNA) oligo containing the consensus TOP2 binding site induced by the TOP2A DNA binding domain (fig. S6B). However, intriguingly, the PNU142586-dsDNA-TOP2A DNA binding domain complex, where biotin-labeled dsDNA oligo is bound, exhibited a distinct gel shift pattern compared to the etoposide-dsDNA-TOP2A DNA binding domain complex, which indicates DNA cleavage status. Furthermore, the PNU142586-dsDNA-TOP2A DNA binding domain complex appeared at a higher position on the gel. These observations from the EMSAs indicate that the conformation of the PNU142586-dsDNA-TOP2A DNA binding domain complex is less compact, suggesting an inability to induce DNA cleavage. Collectively, these results suggest that PNU142586 operates before the cleavage step of TOP2 but does not prevent the initial binding of TOP2 to DNA. Instead, it likely impairs the correct positioning of DNA required for the cleavage step by disrupting key TOP2-DNA interactions.

Overall, our findings demonstrate that PNU142586 acts as a catalytic inhibitor of TOP2 by inhibiting ATP hydrolysis and disrupting essential TOP2-DNA interactions necessary for cleavage, thereby preventing the formation of the TOP2-DNA cleavage complex (fig. S11). However, the latter mechanism appears to be the primary mode of action because it effectively interferes with TOP2-DNA interactions that are crucial for catalytic activity.

### PNU142586 inhibits proliferation of human blood cell lines via TOP2A and TOP2B

**Fig. 6.**
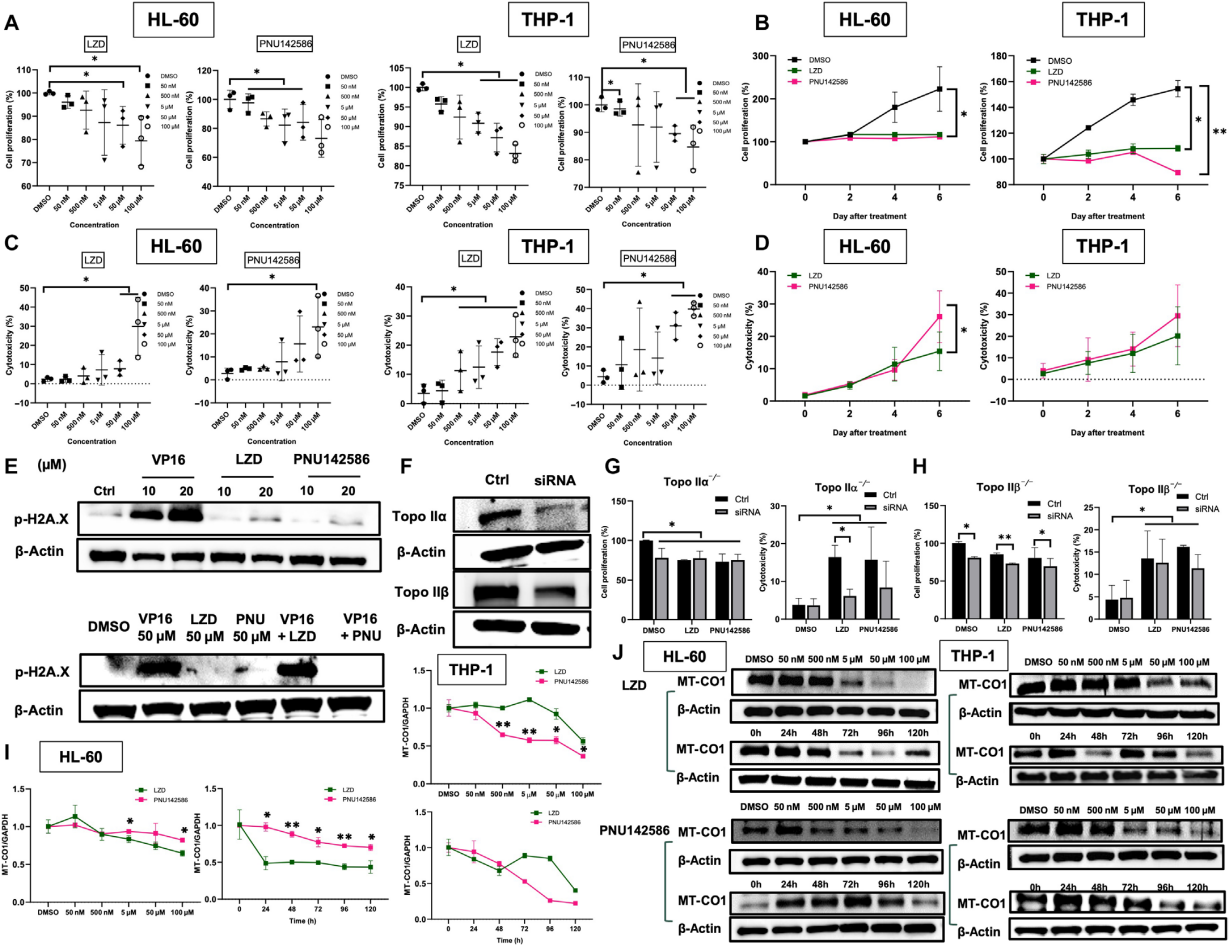
PNU142586 suppresses human blood cell proliferation through TOP2A and TOP2B. HL-60 and THP-1 cells were treated with various concentrations of LZD or PNU142586. After incubation for 144 hours, the cells were directly treated with MTS tetrazolium salt for 2 hours at 37°C to examine proliferation (**A**), or the supernatant was collected for cytotoxicity testing (**B**). The HL-60 and THP-1 cells were then treated with the highest concentration of LZD or PNU142586 (100 μM). At different time points, cells were directly treated with MTS tetrazolium salt for 2 hours at 37°C to examine proliferation (**C**), or the supernatant was collected for cytotoxicity testing (**D**). (**E**) HL-60 cells were treated with DMSO or 10 to 20 μM etoposide (VP16), 10 to 20 μM LZD, or PNU142586 for 2 hours. Immunoblotting measured p-H2A.X levels in the cells, with β-actin as a loading control. The HL-60 cells were then cotreated with 50 μM VP16 and 50 μM LZD or 50 μM PNU142586 for 2 hours. Immunoblotting measured p-H2A.X levels in the cells, with β-actin as the loading control. (**F**) Cells were knocked down for TOP2A and TOP2B using siRNA, and the efficacy was examined using Western blots after 72 hours. The cells were then collected and treated with LZD or PNU142586 to examine cell proliferation and cytotoxicity (**G** and **H**) after 144 hours. Cells were treated with various concentrations of LZD or PNU142586 for 120 hours or the highest concentration at different time points. RNA was then extracted for gene expression analysis (**I**), or MT-CO1 levels were detected using Western blots (**J**). *P < 0.05 and **P < 0.005 versus 0 hours or the DMSO or control group. h, hours.

The inhibitory effects of PNU142586 on TOP2 were evident in a cell-free system. Given that TOP2 is essential for DNA replication, it is widely recognized that most TOP2 inhibitors, including poisons and catalytic inhibitors, induce cytotoxicity (*56*, *58*, *59*). To determine whether PNU142586 affects cell proliferation and/or cytotoxicity in a manner similar to other TOP2 inhibitors, we conducted various cell-based assays using HL-60 promyelocytes and THP-1 monocytes, two well-established human cell lines representative of LZD-induced hematologic toxicity targets. A previous report has linked LZD to impaired cell proliferation via the inhibition of mitochondrial protein synthesis in these cell lines (*25*); hence, LZD was included as a positive control. Our findings revealed that both PNU142586 and LZD suppressed proliferation in HL-60 and THP-1 cells in a dose- and time-dependent manner. Specifically, LZD and PNU142586 exhibited concentration-dependent suppression of cell growth with the highest concentration (100 μM), resulting in a reduction in proliferation of up to 25% compared to the control [dimethyl sulfoxide (DMSO)] (Fig. 6A). Moreover, treatment with maximal concentrations of both LZD and PNU142586 resulted in a slight increase or decrease in proliferation over longer incubation periods, whereas DMSO-induced proliferation significantly and consistently increased over time (Fig. 6B). Conversely, cytotoxicity, as indicated by lactate dehydrogenase levels in the cytosol, escalated in a concentration- and time-dependent manner following treatment with both LZD and PNU142586 (Fig. 6, C and D).

To further investigate the inhibition of cell proliferation in human hematologic cell lines sensitive to PNU142586 and LZD, we used flow cytometry to quantify cells that stained positive for annexin V and propidium iodide, markers of apoptosis and cell death, respectively, following treatment with LZD and PNU142586 at 50 nM and 50 μM (18.47 mg/liter for PNU142586 and 16.87 mg/liter for LZD) for 48 hours. Our results revealed that treatment of LZD and PNU142586, at both 50 nM and 50 μM concentrations, resulted in minimal apoptosis, as indicated by low early and late apoptotic populations comparable to the DMSO control. In contrast, VP16 at 50 μM induced significant apoptosis, with 68.9% of cells in the late apoptotic quadrant, highlighting its pronounced pro-apoptotic activity compared to LZD and PNU142586, which showed negligible apoptotic effects under similar conditions (fig. S7A). Next, to assess the impact of PNU142586 and LZD on the cell cycle, flow cytometry analysis was performed by isolating and staining cell nuclei to determine the distribution of HL-60 cells across different phases of the cell cycle. After 48 hours of treatment, the percentage of cells in the S and G_2_-M phases was slightly higher in the PNU142586-treated group compared to the control group, while treatment with LZD primarily increased the proportion of cells in the S phase (fig. S7B). These results suggest that PNU142586 treatment induces cell cycle arrest at the G_2_-M phase, likely due to its inhibitory effects on cell proliferation (fig. S7B).

For comparison, we examined the effects of VP16, a known topoisomerase II poison, and ICRF-187, a catalytic inhibitor of topoisomerase II. VP16 predominantly induced G_1_ phase arrest, while ICRF-187 caused significant G_2_-M arrest. These findings are consistent with the established mechanisms of these agents. Together, these findings provide valuable insights into the cellular effects of PNU142586, distinguishing its mechanism from that of classical topoisomerase II poisons like VP16. The lack of apoptosis induction was further supported by Western blot analysis of cleaved poly(ADP-ribose) polymerase (PARP) (fig. S7C), indirectly suggesting a distinct mode of action that differs from typical TOP2 poisons. Notably, neither PNU142586 nor LZD caused DNA damage, as evidenced by unchanged phospho-H2AXγ protein levels and pretreatment-antagonized etoposide-induced phospho-H2AXγ protein levels. These results align with our observation that PNU142586 does not stabilize the TOP2-DNA cleavage complex but instead prevents its formation (Fig. 6E).

Given that LZD can be metabolized into PNU142586 through nonenzymatic oxidative reactions or certain cytochrome P450 (CYP) enzymes, such as CYP2J2 and CYP1B1 (*54*, *60*), and the cells were exposed to LZD for an extended period (by 6 days) in our cell-based assays, suppression of proliferation in human blood cell lines following LZD treatment may arise not only from LZD itself but also from PNU142586. We observed an increase in the expression levels of enzymes CYP2J2 and CYP1B1, which are responsible for metabolizing LZD into PNU142586, within 24 hours of treatment in the cell line under investigation. In addition, the amount of LZD was observed to degrade rapidly within the tested cell lines during the first 24 hours, followed by a gradual degradation over the subsequent 120 hours, as quantified by liquid chromatography–mass spectrometry (fig. S7D). These data suggest that the time-dependent influence on cell proliferation and cytotoxicity observed with LZD treatment could be attributed to its metabolites, such as PNU142586.

To ascertain whether the cytotoxicity of PNU142586 in human blood cell lines stems from TOP2 targeting, we conducted the small interfering RNA (siRNA) knockdown of TOP2A and TOP2B using a mixture of three independent siRNAs in HL-60 cells. Western blot analysis confirmed the knockdown of TOP2A and TOP2B post-transfection with siRNA (Fig. 6F). This knockdown of both TOP2A and TOP2B conferred resistance to proliferation and cytotoxicity induced by PNU142586 and LZD. Specifically, LZD and PNU142586 treatment had no effect on cell proliferation and slightly increased cytotoxicity in TOP2A-deficient cells (Fig. 6G). Similarly, TOP2B deficiency significantly attenuated the effects of LZD and PNU142586 on HL-60 proliferation and lactate dehydrogenase levels, although the strength of this influence was lower than that of TOP2A (Fig. 6H). These findings suggest that TOP2A contributes more to the effects of LZD and PNU142586 in HL-60 cells. However, given the slight variation in knockdown efficiency between TOP2A and TOP2B, caution is required in concluding that TOP2A plays a more critical role in PNU142586’s mode of action. Although LZD itself does not inhibit TOP2 activity, its notable contribution to the cytotoxic effects of LZD suggests that a substantial proportion of LZD’s cytotoxicity may be attributed to TOP2 inhibition by its metabolite. Overall, these results suggest that TOP2 is a primary cellular target of PNU142586, contributing to the cytotoxicity observed in human blood cell lines. Furthermore, LZD appears to exhibit cytotoxicity through conversion into PNU142586.

### PNU142586 inhibits mitochondrial gene transcription in human blood cell lines

The mechanism underlying LZD-induced cytotoxicity via the impairment of mitochondrial protein translation is well documented (*25*). Our investigation revealed that PNU142586, the primary metabolite of LZD, catalytically inhibits TOP2 by binding directly to its two domains, thus preventing proliferation and exerting its cytotoxicity without inducing apoptosis or cell death in human blood cell lines. In addition to its role in DNA replication, TOP2 is also involved in gene transcription, particularly in the elongation process (*61*). Pharmacological inhibition of TOP2 can reduce the transcription of genes, leading to lower protein expression. Furthermore, although there is some controversy for the role of TOP2 in mitochondrial gene regulation (*62*), TOP2 is present in both the nucleus and mitochondria, with TOP2B involved in it by altering the topology of mitochondrial DNA (*63*–*65*). On the basis of this, we investigated whether PNU142586 inhibits mitochondrial transcription and protein translation by monitoring the expression of MT-CO1, a protein crucial in mitochondrial complex IV deficiency encoded by the mitochondrial genome (*24*–*26*), in HL-60 and THP-1 cells treated with LZD and PNU142586.

Overall, both LZD and PNU142586 down-regulated *MT-CO1* transcription at high concentrations [above 5 μM (1.85 mg/liter for PNU142586 and 1.69 mg/liter for LZD) for HL-60 and 0.5 μM (0.19 mg/liter for PNU142586 and 0.17 mg/liter for LZD) for THP-1] and for an incubation period of more than 48 hours, although PNU142586 had a more pronounced effect than LZD in THP-1 cells (Fig. 6I). At the protein level, a similar trend was observed, with PNU142586 more strongly suppressing MT-CO1 levels in both HL-60 and THP-1 cells (Fig. 6J). Given that PNU142586 is an inactive metabolite of LZD (*28*, *60*, *66*), it is unlikely to inhibit bacterial and mitochondrial ribosomes. In cell lines treated with LZD, because the stress elicited by the inhibition of mitochondrial translation caused by LZD itself is likely to increase the demand for the transcription of mitochondrial genes, the down-regulation of *MT-CO1* transcription could be caused by PNU142586-mediated TOP2 inhibition. Therefore, the suppression of mitochondrial protein expression may be primarily attributed to transcriptional inhibition by PNU142586 in both cell lines.

In cell lines with the knockdown of TOP2A or TOP2B, the drug-induced suppression of *MT-CO1* expression was not clearly reversed for either PNU142586 or LZD (fig. S7E). This was tentatively attributed to the up-regulation of *MT-CO1* expression following the knockdown of either TOP2A or TOP2B regardless of the treatment used. As previously reported (*67*, *68*), this higher *MT-CO1* expression is potentially linked to the increased activity of alternative topoisomerases, such as DNA topoisomerase 1 in the nucleus (TOP1) and in mitochondria (TOP1MT), when the activity of TOP2A or TOP2B is inhibited. Given that the higher activity of TOP1 and TOP1MT can compensate for the knockdown of TOP2A or TOP2B, the *MT-CO1* expression results observed with TOP2A or TOP2B knockdown can potentially be explained by LZD or PNU142586 inhibiting TOP1 or TOP1MT. Although the inhibitory potency of PNU142586 against TOP1 is much lower than that against TOP2A and TOP2B, it still inhibited TOP1-mediated relaxation at the maximum concentration tested (2.5 mM), while LZD and PNU142300 did not (fig. S7F). Furthermore, considering the high sequence identity and similar biochemical characteristics of TOP1 and TOP1MT (*69*), as well as their response to reference inhibitors such as camptothecin, it is reasonable to speculate that PNU142586 has inhibitory potential against TOP1MT, a key topoisomerase that is exclusively present in mitochondria and unwinds mitochondrial DNA (*63*). These results indicate that PNU142586 exerts cytotoxicity by inhibiting TOP2 activity, not only by directly impeding replication but also by inducing mitochondrial toxicity through the suppression of mitochondrial gene transcription. These observations also indicate that LZD may similarly induce mitochondrial toxicity and subsequent cytotoxic effects because it undergoes intracellular metabolism to produce PNU142586. This mechanism is thus proposed to occur in conjunction with the known pathway involving mitochondrial ribosome inhibition, and the findings of the present study suggest that the *MT-CO1* transcript can be used as a biomarker candidate for the monitoring and prediction of LZD toxicity.

### PNU142586 exhibits in vivo toxicity through the inhibition of TOP2 and suppresses mitochondrial gene transcription

The genus *Xenopus* has been widely used as a vertebrate animal model in molecular and physiological research. This is because *Xenopus* oocytes are large, can be readily manipulated in the laboratory, and offer excellent biochemical tractability, making them particularly useful for reproductive and cell cycle analyses and investigations into drug-induced toxicity (*70*, *71*). Moreover, *Xenopus* oocyte extracts are also a valuable model system for studying DNA replication, including the role of TOP2, because the regulation of DNA replication in these extracts mirrors the cell cycle controls observed in vivo (*72*–*74*).

**Fig. 7.**
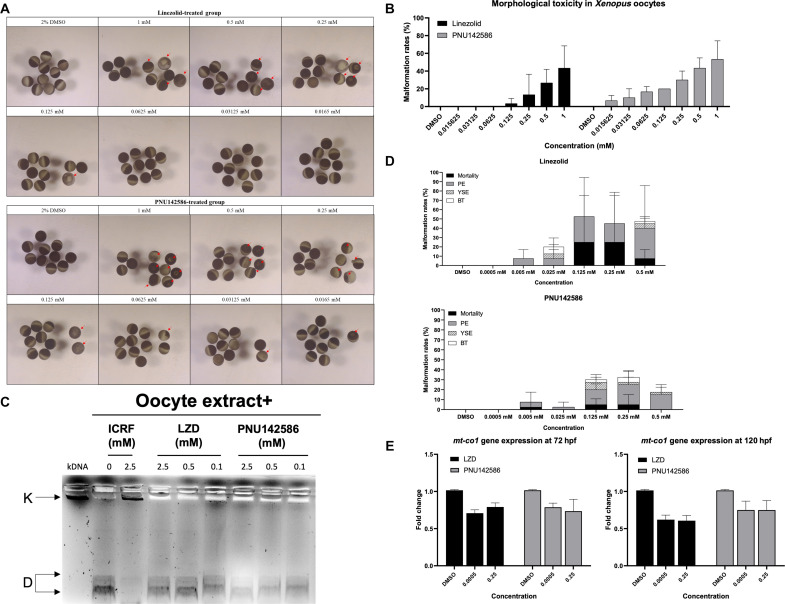
PNU142586 exhibits in vivo toxicity through the inhibition of TOP2 and suppresses mitochondrial gene transcription. (**A**) Effects of LZD and PNU142586 exposure on the morphology of *X. laevis* oocytes over 168 hours. The change in pigmentation in the animal and vegetal poles is indicated by red arrows. DMSO (2%) was used as the control. Scale bar, 500 μm. (**B**) Malformation rate for the *X. laevis* oocytes (*n* = 30; 10 oocytes in triplicate for each group). Means ± SD. (**C**) The kNDA decatenation activity for *Xenopus* oocyte extract was measured in the presence of LZD or PNU142586 (in mM). Novobiocin (in mM) was used as the positive control. (**D**) Effects of LZD and PNU142586 exposure on the development of zebrafish larvae. Morphological abnormalities were observed daily from treatment at 24 to 120 hpf. DMSO (0.1%) was used as the control (*n* = 40; 10 larvae in quadruplicate for each group). Means ± SD. PE, pericardial edema; YSE, yolk sac edema; BT, bent tail. (**E**) Expression analysis of the *mt-co1* gene in LZD- and PNU142586-treated zebrafish larvae at 72 and 120 hpf. RNA was extracted from the treated larvae after imaging (*n* = 30; 10 larvae in triplicate for each group). Means ± SEM.

To validate our in vitro findings of PNU142586-induced toxicity through TOP2 inhibition in a simplified in vivo setting, we used *Xenopus* oocytes. The impact of LZD and PNU142586 on oocyte survival was assessed on the basis of morphological changes. *Xenopus* oocytes were exposed to LZD at final concentrations of 1 mM (337.35 mg/liter), 0.5 mM (168.68 mg/liter), 0.25 mM (84.19 mg/liter), 0.125 mM (42.17 mg/liter), 0.0625 mM (21.084 mg/liter), 0.003125 mM (10.54 mg/liter), and 0.015625 mM (5.27 mg/liter); and to PNU142586 at final concentrations of 1 mM (369.34 mg/liter), 0.5 mM (184.67 mg/liter), 0.25 mM (92.34 mg/liter), 0.125 mM (46.17 mg/liter), 0.0625 mM (23.08 mg/liter), 0.003125 mM (11.54 mg/liter), and 0.015625 mM (5.77 mg/liter). Exposure of *Xenopus* oocytes to LZD and PNU142586 over 168 hours resulted in significant changes in pigmentation at the oocyte animal pole and induced a toxic effect on the oocytes, indicated by a bleaching phenotype that was not observed in the DMSO-treated control (Fig. 7A). Both LZD and PNU142586 treatments led to a dose-dependent decrease in the proportion of morphologically normal oocytes. PNU142586 induced a toxic effect on the oocytes at substantially lower concentrations than LZD [15 or 62.5 μM (5.54 or 23.08 mg/liter) for PNU142586 compared to 125 or 500 μM (42.17 or 168.68 mg/liter) for LZD] and caused a higher proportion of oocyte toxicity at the same concentration [500 μM (184.67 mg/liter for PNU142586 and 168.68 mg/liter for LZD); ~43% for PNU142586 compared to ~27% for LZD] (Fig. 7B). In addition, to determine whether PNU142586 inhibits TOP2 in *Xenopus* oocytes, we conducted kDNA decatenation assays using *Xenopus* oocyte extract. The assays revealed that PNU142586 dose-dependently inhibited kDNA decatenation, with around half of the activity observed at 500 μM, while LZD did not exhibit any inhibitory effect (Fig. 7C).

The discrepancy in the toxicity levels of PNU142586 and LZD observed in *Xenopus* oocytes, which was not observed in the cell line experiments, revealed that the toxicity of PNU142586 is more potent. This can be attributed to differences in the expression profiles of the metabolizing enzymes. Enzymes such as CYP2J2 and CYP1B1, which convert LZD to PNU142586, exhibited robust expression in the cell lines under investigation. However, it is thought that these enzymes might exhibit lower expression and, thus, PNU142586 metabolized from LZD would be lower in *Xenopus* oocytes, presenting the difference in inherent toxic potential of PNU142586 and LZD. Intriguingly, while maternal genes expressed in animal oocytes remain highly conserved across species, CYP enzymes including CYP2J2 and CYP1B1 are not (*75*). Thus, *Xenopus* oocyte systems are more useful for clarifying the toxic effects of PNU142586 relative to LZD when compared with cell line systems.

The zebrafish (*Danio rerio*) has also emerged as a valuable animal model for investigating toxicology and disease mechanisms in scientific research and has been widely used for high-throughput, in vivo screening of new drug candidates, including antimicrobials (*76*–*78*). To assess the acute toxicity of LZD and PNU142586 in vivo, we exposed groups of 10 zebrafish embryos to concentrations ranging from 0 to 0.5 mM. The toxic effect of LZD and PNU142586 on the zebrafish embryos was evaluated on the basis of observed morphological changes and mortality rates. Exposure to both LZD and PNU142586 resulted in significant morphological changes, particularly pericardial edema, compared to the DMSO-treated control group at concentrations of 0.125 mM or higher and after 72 hours postfertilization (hpf) (Fig. 7D, fig. S8, and table S4). Both compounds exhibited a dose-dependent increase in the proportion of embryos exhibiting pericardial edema. In PNU142586-treated embryos, pericardial edema appeared earlier (at 72 hpf) compared to those treated with LZD but exhibited a tendency toward resolution after 96 hpf. Conversely, in the LZD-treated group, pericardial edema occurred at a higher incidence at 96 hpf compared to PNU142586-treated embryos and persisted until 120 hpf or led to mortality.

To confirm the suppression of mitochondrial transcription by PNU142586 in vivo, the expression of *MT-CO1* was analyzed using real-time quantitative polymerase chain reaction (RT-qPCR) (Fig. 7E). Compared to the control group, *MT-CO1* expression was significantly down-regulated at concentrations above 0.5 μM (0.19 mg/liter for PNU142586 and 0.17 mg/liter for LZD) in both PNU142586- and LZD-treated embryos, which was lower than the concentration that led to morphological changes in the embryos. The down-regulation of *MT-CO1* transcription was consistent across the concentrations tested, with no clear concentration-dependent decrease. However, the *MT-CO1* expression patterns differed between LZD and PNU142586 over time. In LZD-treated embryos, *MT-CO1* expression decreased from 72 to 120 hpf, while it remained stable during this period in PNU142586-treated embryos. These results differ from our cell-based assays, where *MT-CO1* transcription decreased in a concentration- and time-dependent manner for both compounds, correlating with MT-CO1 protein expression and cellular phenotypes such as antiproliferation and cytotoxicity. The discrepancy may arise because *MT-CO1* expression in zebrafish was measured at the individual level, rather than in specific organs or tissues, thus potentially obscuring clear associations with the drug concentration and time. These findings suggest that other nuclear or mitochondrial genes regulated by TOP2 as a biomarker need to be identified because they may more strongly correlate with or be sensitive to toxicity phenotypes at the in vivo level. In addition, the differing toxicity patterns observed over time between PNU142586 and LZD in zebrafish embryos may be due to distinct mechanisms of toxicity, including differences in mitochondrial ribosome inhibition and the suppression of DNA replication and transcription via TOP2 inhibition. In addition, unique metabolic adaptations in embryos may contribute to these differences in the responses. Overall, despite some discrepancies, our in vivo findings are consistent with our in vitro observations, confirming the toxic effects of PNU142586 and LZD and verifying the in vivo relevance of our mechanistic studies.

## DISCUSSION

In the present study, we identified the primary targets of PNU142586, the principal metabolite of LZD, as TOP2A and TOP2B at the molecular, cellular, and in vivo levels. This investigation was prompted by clinical data indicating that elevated exposure to PNU142586 is associated with an increased risk of LZD-induced toxicity, even in patients without renal impairment. PNU142586 exerts its inhibitory effects on replication and transcription by hindering DNA binding to TOP2A and TOP2B with a favorable conformation that induces cleavage and by inhibiting ATP hydrolysis. This action leads to antiproliferative and toxic effects on target cells or organs, furthering the understanding of LZD-induced hematologic adverse events. These findings can not only guide the design of safer oxazolidinone antibiotics but also offer a potential biomarker for monitoring the safety of LZD in clinical practice, facilitating patient stratification and the development of rational dosing regimens.

LZD is approved for clinical use as an oxazolidinone antibiotic for the treatment of MDR-TB and drug-resistant Gram-positive pathogens (*4*, *6*, *79*). Despite its efficacy, prolonged usage is linked to markedly higher incidences of severe hematologic adverse events, such as thrombocytopenia and anemia (*5*, *17*, *20*, *79*). While several mechanisms have been proposed to explain these events (*4*, *5*, *17*, *24*), none of these completely account for the diverse array of observations that have been reported, which include the involvement of LZD metabolites (*17*, *28*) and the disparity between mitochondrial protein synthesis inhibition and clinical safety profiles observed in certain oxazolidinones (*24*, *25*, *80*–*83*). This highlights the need to identify other molecular mechanisms to explain these observations for LZD and other oxazolidinones, which includes determining the exact molecular targets of LZD metabolites, to promote the development of safer LZD alternatives.

Our study suggests TOP2A and TOP2B as the primary targets of PNU142586. This finding is particularly noteworthy, as clinical data have demonstrated a link between higher levels of PNU142586 and an increased risk of LZD-induced toxicity, regardless of renal function. In particular, PNU142586 inhibits TOP2-mediated kDNA decatenation and scDNA relaxation in cell-free systems while also demonstrating antiproliferative activity, cytotoxicity, and the reversal of PNU142586-induced cellular responses following TOP2 knockdown in human blood cell lines. Our investigation also revealed that PNU142586 had a concentration-dependent in vivo toxic effect on *Xenopus* oocytes and zebrafish embryos. A previous study has emphasized the importance of TOP2 in resolving DNA topological issues to maintain replication and transcription fidelity (*61*). As such, pharmacological inhibition of TOP2 leads to the suppression of gene replication and transcription, thus impairing proliferation and global toxicity. Similar to PNU142586, most TOP2 inhibitors have been reported to lead to cytotoxicity, antiproliferative activity, and dose-dependent hematologic adverse events (*43*, *44*). While typical TOP2 inhibitors, such as TOP2 poisons, are known to induce cell cycle arrest and apoptosis, our findings revealed distinct mechanisms of action for PNU142586. Specifically, PNU142586 inhibits proliferation and induces cytotoxicity by arresting the cell cycle predominantly at the S and G_2_-M phases. This suggests that the mechanisms underlying the effects of PNU142586 differ from those of classical topoisomerase II poisons, such as VP16. There are various catalytic points at which TOP2 can be inhibited, and some TOP2 catalytic inhibitors, such as ICRF-187 and HU-331, exhibit similar cellular characteristics to PNU142586 (*84*, *85*). Moreover, the notable transcriptional impairment of MT-CO1, a mitochondrial gene, was observed in human blood cell lines and a zebrafish model treated with PNU142586 in the present study, providing indirect evidence for a TOP2-mediated mechanism and enhancing the current understanding of mitochondrial toxicity in LZD-induced hematologic adverse events.

Our findings also address the perplexing lack of correlation between the extent of mitochondrial ribosome inhibition and the safety profiles observed in preclinical and clinical investigations of LZD and other oxazolidinones such as tedizolid and sutezolid, a phenomenon that cannot be explained by the direct inhibition of mitochondrial protein synthesis. Structurally, tedizolid differs from LZD in that its morpholine ring is replaced with a tetrazole-fused pyridine group (fig. S1) (*86*). This substitution fundamentally alters its metabolic pathway, as tedizolid cannot produce toxic metabolites such as PNU142586 that are derived from the breakdown of the morpholine ring in LZD. Previous studies have noted that tedizolid exhibits greater potency in inhibiting mitochondrial protein synthesis compared to LZD, presumably due to the presence of additional interaction sites in mitochondrial ribosomes (*80*, *87*, *88*). Intriguingly, despite its stronger inhibition, tedizolid demonstrates a more favorable safety profile than LZD in preclinical and clinical settings, although limited clinical data are available (*81*–*83*). Notably, both human and animal studies have revealed that the administration of a tedizolid phosphate prodrug results in tedizolid being the sole detectable metabolite in plasma (*89*–*91*), distinguishing it from LZD, which can be broken down into detectable metabolites such as PNU142586. Our investigation further demonstrated that tedizolid lacked inhibitory activity for both TOP2A and TOP2B in in vitro enzyme assays (data not shown). Assuming that LZD-induced toxicity stems not only from direct mitochondrial ribosome inhibition via LZD itself but also from TOP2 inhibition because of its major metabolite PNU142586, tedizolid’s capacity to avoid TOP2 inhibition while selectively inhibiting mitochondrial ribosomes leads to reasonable predictions of weaker toxic effects.

Similarly, sutezolid, another oxazolidinone, shares structural similarities with LZD but exhibits key differences. Sutezolid is a structural analog of LZD, differing only by the substitution of the oxygen atom in the morpholine ring with a sulfur atom, resulting in a thiomorpholine ring (fig. S1) (*92*). This structural modification prevents the formation of metabolites such as PNU142586 and instead leads to the generation of a sulfoxide-containing metabolite, PNU101603, which retains the intact thiomorpholine ring (fig. S1) (*93*, *94*). Sutezolid exhibits similar potency to LZD in inhibiting mitochondrial protein synthesis, which contrasts with the greater potency observed for tedizolid (*20*). Moreover, PK studies reveal that sutezolid achieves a steady-state AUC more than three times higher than LZD, indicating superior drug exposure (*95*). In single ascending dose and multiple ascending dose PK studies, as well as in a phase 2 early bactericidal activity study, sutezolid did not result in serious adverse events, including hematologic adverse events such as myelosuppression (*94*, *96*–*98*). Similar to tedizolid, this improved safety profile highlights the absence of metabolites that inhibit TOP2, such as PNU142586, in its metabolic pathway. Collectively, these findings underscore that, in addition to the direct inhibition of mitochondrial ribosome activity, LZD-induced hematologic adverse events are notably influenced by TOP2 inhibition mediated by its metabolite PNU142586. The structural modifications in tedizolid and sutezolid, which prevent the formation of this toxic metabolite, provide a plausible explanation for their improved safety profiles despite their similar or even greater potency in mitochondrial protein synthesis inhibition.

Our study thus complements the hypothesis that mitochondrial toxicity is a primary contributor to the hematologic adverse events associated with LZD rather than contradicting it. As previously discussed, PNU142586, either directly absorbed from circulating blood or produced within target cells through prolonged exposure to LZD, can lead to the suppression of gene transcription. This results in lower protein production, including mitochondrial proteins, potentially exacerbating LZD-induced mitochondrial toxicity. In addition, we cannot discount the involvement of other mechanisms in LZD-induced hematologic adverse events because our investigation, initiated with an in silico inverse docking study and chemically induced transcriptomic signature analysis, was constrained by the databases used, which may not cover all potential target proteins or perturbagens. Although the inhibitory potency of PNU142586 against TOP1 is not as strong as against TOP2, we identified TOP1 as another target protein of PNU142586 in our subsequent experiments. There is a possibility that other metabolites derived from PNU142586 or PNU142300 via hepatic or target cell metabolism may exhibit greater inhibitory activity against TOP2 through more robust binding interactions (fig. S1).

Our research identified the molecular mechanisms through which PNU142586 acts to inhibit TOP2. First, we identified PNU142586 as a catalytic inhibitor of both TOP2A and TOP2B using cell-free and cell-based assays. This conclusion was based on evidence that PNU142586 failed to trap the covalent TOP2-DNA cleavage complex in TOP2-mediated DNA cleavage assays, did not induce cell death or apoptosis in flow cytometry analysis, and did not trigger DNA damage as reflected by phospho-H2AXγ protein levels in human blood cell lines. Our investigation also revealed that, in addition to its inhibition of ATP hydrolysis, PNU142586 prevented DNA from binding to TOP2 in a favorable conformation for cleavage, thus inhibiting its catalytic activity. This was supported by results from ethidium bromide displacement assays and EMSAs, which demonstrated that PNU142586 did not intercalate into DNA nor interfere with TOP2-DNA binding.

Although our data revealed direct physical interactions between PNU142586 and both the ATPase and DNA binding domains of TOP2, ATP replacement assays revealed that PNU142586 blocked TOP2 in a relatively ATP-insensitive manner. Therefore, we propose that the main mode of action of PNU142586 is hindering the binding of DNA with a favorable conformation for cleavage (fig. S11). Our in silico docking studies also suggested that PNU142586 may interfere with the favorable binding of DNA to TOP2 by sterically hindering the DNA backbone near the ligand docking pocket in the initial status, as well as by inducing conformational changes in the DNA binding domain. This mechanism may involve allosteric communication between the different domains of TOP2 because subtle changes in the DNA binding domain can propagate to the ATPase domain and vice versa. It is widely accepted that different regions of TOP2 cooperatively modulate drug sensitivity, raising the possibility of synergistic inhibition through simultaneous or consecutive binding of compounds to both the ATPase and DNA binding domains (*99*, *100*). Despite its relatively weak affinity for both the ATP and DNA binding domains, PNU142586 demonstrated similar inhibitory activity to the reference compounds for TOP2 decatenation. This suggests a simultaneous or cooperative mode of action for PNU142586, which provides valuable insights into TOP2 molecular biology and can assist in the discovery of novel TOP2 inhibitors.

The observed IC_50_ value of PNU142586 for TOP2 inhibition (~150 μM) in cell-free assays may appear modest; however, this value is comparable to that of novobiocin, a well-characterized TOP2 catalytic inhibitor, under the same experimental conditions (fig. S9). This comparison underscores the pharmacological relevance of PNU142586’s inhibitory activity against TOP2. Moreover, the mechanism of action of some catalytic inhibitors, such as PNU142586, often involves preventing the DNA-damaging activity of TOP2 poisons. The ability to interfere with such activity may better represent the potency of catalytic inhibitors. In our cell-free system, PNU142586 effectively inhibited the DNA cleavage activity of etoposide—a clinically used TOP2 poison—at comparable concentrations, as shown in Fig. 4C. This finding suggests that, despite its moderate IC_50_ value, PNU142586 exhibits notable biological activity by disrupting key TOP2-mediated processes. In addition, in cell-based assays, PNU142586 suppressed etoposide-induced DNA damage at concentrations similar to those of etoposide (Fig. 6E). These results reinforce the hypothesis that PNU142586 functions within a biologically relevant concentration range to disrupt TOP2 activity. Furthermore, the activity of PNU142586 may be amplified in cellular systems because of the inherent complexity of the cellular context, which enhances TOP2 inhibitory effects, combined with its ability to inhibit both TOP2A and TOP2B. In particular, this dual inhibition disrupts TOP2-specific processes, such as DNA catenation and knotting resolution, which are essential for maintaining chromosomal integrity and cannot be fully compensated by alternative cellular mechanisms. Collectively, these data support the pharmacological relevance of PNU142586’s activity against TOP2, particularly within the complex physiological environment of cellular systems.

In the present study, PNU142586 exhibited a comparatively stronger inhibitory impact in cell-based assays that tested both cell proliferation and cytotoxicity than in enzyme assays using recombinant TOP2A or TOP2B. This difference can be attributed, in part, to the inherent complexity of the cellular context, which enhances the apparent inhibitory potency of PNU142586. The observed IC_50_ value of PNU142586 in the enzyme-based assay (~150 μM) reflects the simplified conditions of the recombinant protein system, where the complexity of the cellular environment is absent. In this system, essential cellular interactions, cofactors, and physiological processes that amplify TOP2 inhibition are not present, leading to higher concentrations being required to observe notable inhibition. In contrast, cellular assays inherently capture the physiological relevance of TOP2 inhibition. In these systems, TOP2A and TOP2B play indispensable roles in DNA replication and cell division. The disruption of these critical functions likely amplifies the cytotoxic effects of PNU142586, even at lower concentrations. This inherent complexity of the cellular environment provides a plausible explanation for the stronger inhibitory effects observed in cellular assays compared to enzyme-based assays. In addition, it appears most likely that PNU142586 exerts its effects by codominantly suppressing both TOP2 paralogs within the cell, as our cell lines expressed both TOP2A and TOP2B. The inhibition of TOP2 activity by PNU142586 likely prevents DNA catenation and knotting resolution, which are unique functions of TOP2 that cannot be compensated for by other enzymes. This process may more effectively enhance the suppression of cell proliferation signaling. Moreover, our choice of proliferating cell lines, such as leukemia cell lines, may contribute to the observed sensitivity to TOP2 inhibitors, given TOP2’s vital role in DNA replication termination and chromosome segregation during mitosis.

In the in vivo zebrafish model, PNU142586 exhibited a dose-dependent increase in the proportion of embryos with toxic phenotypes. However, its effect on *MT-CO1* gene expression did not show a clear dose dependency. This discrepancy can be attributed to measuring the transcripts for the whole embryos rather than for specific target organs or tissues. However, these differing results have other underlying implications. Considering that mitochondrial transcription encompasses nearly the entire genome in polycistronic form and that most genes on the same strand of the mitochondrial genome are transcribed with equal efficiency because of its structural and transcriptional organization (*101*, *102*), these results suggest that nuclear genes regulated by TOP2 might serve as more sensitive biomarkers for predicting antiproliferation or global toxicity in vivo. This observation is consistent with findings in different cell systems, where ribosome-targeting antibiotics, including LZD, suppress mitochondrial protein production without directly correlating with an antiproliferative phenotype, as seen in mouse T helper 17 cells (*103*). Therefore, further in-depth investigations, including those that use untargeted approaches, are needed to validate this hypothesis and to identify a more reliable biomarker, or a combination of biomarkers, alongside *MT-CO1* for predicting LZD-induced toxicity.

The pharmacologically relevant concentration of PNU142586 in humans, such as the predicted steady-state average concentration [~30 μM (11.08 mg/liter)] (table S5), falls within but toward the lower end of the concentration range that demonstrates significant antiproliferative effects in human blood cell lines [a ~25% reduction in proliferation at 100 μM (36.93 mg/liter) and a 10 to 20% reduction at 10 μM (3.69 mg/liter)] (*31*, *60*, *66*). However, the limited number of PK studies on PNU142586 and their small sample sizes may lead to potential inaccuracies in PK predictions. Notably, recent population PK modeling efforts have substantially underpredicted concentrations of PNU142586, particularly at higher concentrations (*31*). Furthermore, the time-dependent nonlinear PK of LZD complicates the prediction of concentration levels (*104*–*106*), especially during prolonged exposure when toxicity may occur. In addition, the tissue penetration of LZD and PNU142586 influences their concentrations in target tissues such as bone marrow. Although this remains poorly characterized, one clinical study indicated that the exposure of LZD in bone marrow is not substantially different from that in plasma (*107*). Consequently, assuming that PNU142586 has similar tissue penetration characteristics based on its structure, we speculate that the concentration of PNU142586 in target tissues may adequately overlap with the effective concentration range exhibiting toxicity found in our study. Nonetheless, these assumptions warrant further investigation in future research.

The need for novel therapeutics to combat tuberculosis has increased because of the increasing emergence of resistant strains such as MDR-TB. Collaborative efforts between the public and private sectors in drug discovery and clinical development have yielded promising candidates for treatment, including new compounds within the oxazolidinone class. A major challenge lies in identifying host molecular targets for the most potent drugs that induce toxicity, with the aim of addressing safety concerns while exploring innovative chemotypes. In this regard, gaining insights into the mode of action of LZD-induced hematologic toxicity in the present study offers a fresh perspective for the design of new oxazolidinone class drugs and potential drug combinations. This approach can thus mitigate safety concerns associated with these drugs and enhance their therapeutic potential.

## MATERIALS AND METHODS

### Clinical PK study

#### Ethical approval

The study was performed according to the Declaration of Helsinki and was approved by the Institutional Review Boards (IRB) of the Center for Personalized Precision Medicine of Tuberculosis (cPMTb; Inje University College of Medicine, Busan, Republic of Korea) and Dr. Soetomo General Academic Hospital (Surabaya, Indonesia) (IRB no. 4/E1/KP.PTNBH/2021; clinical trial no. NCT05280886). All patients provided written informed consent. The material transfer agreement between Inje University and Universitas Airlangga was approved by the Ministry of Health Indonesia (material transfer agreement no. HK.07.01/I/2481/2022).

#### Patients and study design

This study was part of an international prospective observational cohort study aiming to develop personalized pharmacotherapy for patients with tuberculosis. Herein, we prospectively enrolled patients with tuberculosis on an LZD-based regimen for at least 2 weeks to achieve a steady-state concentration. Blood samples were randomly collected within 0 to 24 hours after the last dose of LZD and stored in heparin tubes in the Clinical Pathology Laboratory of Dr. Soetomo General Academic Hospital. One and up to two samples were obtained from outpatients and inpatients, respectively. Blood samples were centrifuged at 2000*g* at 4°C for 10 min. Plasma was extracted immediately and stored at −80°C until analysis.

#### Measurement of concentrations of LZD and its metabolites

The concentrations of LZD, PNU142300, and PNU142586 in plasma were determined using a validated liquid chromatography–tandem mass spectrometry method. For protein precipitation, plasma samples were added to acetonitrile. The supernatant was then diluted with water containing 0.1% formic acid. Chromatographic separation was performed on a C18 column using gradient elution with mobile phases consisting of water containing 0.1% formic acid and acetonitrile containing 0.1% formic acid. The calibration ranges were 0.4 to 20 mg/liter for LZD, 0.1 to 5 mg/liter for PNU142300, and 0.2 to 10 mg/liter for PNU142586. The correlation coefficients (*R*^2^) were greater than 0.9953 for all analytes. The intraday and interday accuracy ranged from 95.5 to 111.6% for LZD, 98.4 to 101.6% for PNU142300, and 98.8 to 106.0% for PNU142586. Precision was less than 4.0% for LZD, less than 8.6% for PNU142300, and less than 4.3% for PNU142586.

#### Bayesian estimation of individual PK parameters and concentration-time profile prediction

In this study, we used a Bayesian estimation approach to estimate individual PK parameters for each patient using the reported population PK model (*31*) in conjunction with the measured drug concentrations. Specifically, we used the population PK model parameters as priors and combined them with the observed concentration data from each patient to estimate the individual PK parameters. This approach allowed for the integration of population-level information with individual patient data, thereby providing more accurate and personalized PK profiles. Following the estimation of individual PK parameters, we predicted the concentration-time profiles for each patient based on their estimated parameters. This method enhances the precision of PK predictions by accounting for both interindividual variability and specific patient characteristics. Outlier patients with excessively high or low concentrations were excluded to avoid overfitting the model to individual predictions. This methodology enabled us to assess drug exposure and its association with toxicity outcomes more accurately in the clinical cohort.

#### Data analysis

AUC was calculated from the predicted concentration-time profiles for each patient using the trapezoidal rule. Linear regression and statistical significance were analyzed using R version 4.3.2 (https://cran.r-project.org/). A *P* value of ≤0.05 was considered indicative of a statistically significant difference in AUC between patients with and without LZD-induced toxicity, as determined by the Wilcoxon rank-sum test.

### Inverse virtual screening using SePreSA and ACID

The SePreSA server, a web-based tool for inverse docking, was developed to predict drug targets based on a collection of structural models of nearly all well-known serious adverse drug reactions (*40*). On the server, potential targets of a ligand can be obtained by submitting a Mol2 ligand file. The SMILE codes for LZD, PNU14230, and PNU142586 were retrieved from PubChem and converted to Protein Data Bank (PDB) format using CORINA. The minimal energy conformations of the chemicals were then simulated, and the charge and hydrogens of the proteins and chemicals were added using VEGA ZZ. OPENBABEL was used to convert PDB format back to Mol2 format. The relative ligand-receptor interaction strength was assessed using a two-directional *Z*-transformation scoring algorithm and then ranked to predict the potential targets.

The ACID server, a comprehensive web platform with a user-friendly interface, was designed on the basis of a consensus inverse docking strategy to substantially reduce the time required to gather and analyze data without human intervention (*42*). The SMILE codes of LZD, PNU14230, and PNU142586 were retrieved from PubChem and converted to PDB format using CORINA. The minimal energy conformations of the chemicals were then simulated, and the charge and hydrogens of the proteins and chemicals were added using VEGA ZZ. OPENBABEL was used to convert PDB format to structure data file (SDF) format, and this was submitted to the server. The interaction energy in ligand-receptor complexes was assessed on the basis of the sum of the changes in the binding energy or docking score of the poses.

### Transcriptomic signature analysis using SigCom LINCS

The SigCom LINCS tool (https://maayanlab.cloud/sigcom-lincs) is a web-based platform that facilitates the analysis and comparison of more than 1.5 million gene expression signatures derived from sources such as LINCS, GTEx, and GEO. In our study, we input “linezolid” as the perturbagen, “blood” as the tissue, and “THP-1” as the cell line to search for relevant signatures. From the generated data, transcriptomic signatures corresponding to clinically relevant concentrations of LZD (10 and 3.3 μM) were selected. These signatures were then used to perform a signature similarity search within SigCom LINCS to identify drugs with transcriptomic profiles closely resembling those induced by LZD. The analysis focused on the top 10 drugs with the highest similarity scores based on *z*-scores from the LINCS L1000 Chemical Perturbations (2021) database.

### Targeted molecular docking of LZD and its metabolites with the ATPase domain and DNA binding domain of TOP2A

The molecular docking of LZD and its metabolites PNU14230 and PNU142586 with the ATP binding site of the ATPase domain of TOP2A was assessed using AutoDock Vina. Similar to the inverse virtual screening, data in PDB format for LZD and its metabolites were used for targeted docking. The crystal structures of the ATPase domain (1zxm) and the DNA binding domain (5gwk) of TOP2A were pretreated using PyMOL (www.pymol.org) for the removal of water molecules, heteroatoms, ions, and original ligands and the addition of polar hydrogen atoms. The ligand binding site was defined using the reference ligand AMPPNP for the ATPase domain and the reported potential drug-binding pocket for the DNA binding domain. The geometric center of the reference ligand or the core amino acid of the drug-binding pocket in the structure was set as the center of the enclosing box (30 Å by 30 Å by 30 Å) using the AutoDock Vina plug-in in PyMOL. The dockings for virtual screening with AutoDock Vina were conducted according to the standard protocol and parameters recommended by the developers, with “exhaustiveness” set to 16. The molecular interactions for the top-posed protein-ligand complexes were visualized using PyMOL.

### MD simulations

All of the MD simulations were conducted using the GROMACS 2020.6 simulation package using the gromos54a7 force field. In total, four simulations were run, one with the free ATPase domain of TOP2A and one each for LZD and its two metabolites bound to this domain. The ligands, LZD, PNU142586, and PNU142300, were parameterized using the CHARMM General Force Field (CGenFF) to ensure compatibility with the CHARMM36 force field used for the protein. The following steps were performed: The three-dimensional (3D) structures of the ligands were generated and optimized in the most stable conformation using Avogadro. The structure was uploaded to the CGenFF web server (https://cgenff.com) to obtain topology (.itp) and parameter (.prm) files. The CGenFF topology and parameter files were converted to GROMACS-compatible formats (.itp and .gro files) using the CGenFF-GROMACS conversion Python script. The final ligand topology and parameter files are provided in the Supplementary Materials. The protein was protonated at the default pH of 7.0 inside a rhombic dodecahedron box. The explicit simple point charge water model was used to solvate all of the systems with NaCl to render them electrically neutral. Energy minimization was conducted using the steepest descent integrator to fix any inappropriate geometries. The systems were then subjected to 100-ps NVT equilibration using the velocity rescale thermostat method with a temperature coupling time constant of 0.1 ps at 300 K. The systems were equilibrated using NPT, with a velocity rescale thermostat and Parrinello-Rahman barostat algorithm (*108*) implemented for temperature and pressure coupling, respectively. Last, a 50-ns production run was carried out with a continuation of NPT equilibration under periodic boundary conditions with the protein only or the protein-ligand complex and a solvent composed of water and ions separately coupled. During the production run, the system temperature was maintained using a velocity rescale at 300 K, with a coupling time constant of 0.1 ps, and the pressure was coupled using an isotropic Parrinello-Rahman barostat at 1 bar with a coupling constant of 2 ps and a compressibility of 4.5 × 10^−5^ bar^−1^. To calculate the short-range coulombic and van der Waals interactions, the cutoff distance was set to 10 Å with a Fourier grid spacing distance of 1.6 Å. Long-range electrostatic interactions were determined using the particle mesh Ewald algorithm (*109*). All bond lengths were constrained using the linear constraint solver algorithm (*110*). Analysis and plotting were conducted using the GROMACS analysis suite and QtGrace.

### Cryo-EM analysis

The purified TOP2A DNA binding domain was concentrated to 1 mg/ml. Four microliters of protein sample was applied on the 200-mesh holey carbon grids with a hole size and spacing of 1.2/1.3-μm Cu grid (Quantifoil) that has been freshly hydrophilized for 60 s using a glow discharger (Pelco). The grid was then immediately plunge frozen using a Vitrobot Mark VI (Thermo Fisher Scientific). Frozen grids were first screened on a Glacios microscope operating at 200 kV (Thermo Fisher Scientific), and then data were acquired on a Titan Krios (Thermo Fisher Scientific) equipped with a Falcon 4i detector. Data were collected automatically using EPU software (Thermo Fisher Scientific). Cryo-EM image processing was performed using cryoSPARC version 3.1.4 (*111*). Motion correction and contrast transfer function estimation were performed to estimate the quality of the micrographs. Particles were initially picked using the blob picker with a particle diameter of 150 Å. Subsequent 2D classification yielded reference 2D classes, which can be used as an input for a template picker. A total number of 1,221,342 particles are picked using a template picker, and subsequent 2D classifications resulted in 234,556 particles with good alignment and moderate feature of the TOP2A DNA binding domain. 3D classification was conducted ab initio. A good reference model and two bad reference models were generated, and following heterogeneous refinement classified 124,337 particles in the best class. Following nonuniform refinement was performed to generate a high-resolution map. Resolution was estimated using the 0.143 criteria of the gold-standard Fourier shell correlation. All structural figures were prepared using ChimeraX_techpreview.

### In vitro TOP2 activity assays

#### kDNA decatenation assays: TOP2A enzyme

Two units of the TOP2A enzyme were incubated with 200 ng of kDNA in reaction buffer A [containing 50 mM tris-HCl (pH 8.0), 150 mM NaCl, 10 mM MgCl_2_, 0.5 mM dithiothreitol (DTT), and bovine serum albumin (30 μg/ml)], 1 mM ATP in the presence of novobiocin as a control inhibitor, and LZD, PNU142586, or PNU142300 to a final volume of 20 μl. The reaction was conducted on ice and then incubated at 37°C for 30 min before being stopped by adding 5 μl of 5× stop buffer (5% Sarkosyl, 0.125% bromophenol blue, and 25% glycerol). The reaction was resolved using electrophoresis on 1% agarose gel with RedSafe (nucleic acid staining solution) at 135 V for 30 min. The DNA bands were visualized using the ChemiDoc Imaging System (Bio-Rad).

#### kDNA decatenation assays: TOP2B enzyme

First, 1.25 units of the TOP2B enzyme were incubated with 200 ng of kDNA in a reaction assay buffer [containing 5 mM tris-HCl (pH 7.5), 12.5 mM NaCl, 1 mM MgCl_2_, 0.5 mM DTT, and albumin (10 μg/ml)], 0.1 mM ATP in the presence of novobiocin as a control inhibitor, and LZD, PNU142586, or PNU142300 to a final volume of 30 μl. The reaction was conducted on ice and then incubated at 37°C for 30 min before being stopped by adding 5 μl of 5× stop buffer (5% Sarkosyl, 0.125% bromophenol blue, and 25% glycerol). The reaction was resolved using electrophoresis on 1% agarose gel with RedSafe at 135 V for 30 min. The DNA bands were visualized using the ChemiDoc Imaging System (Bio-Rad).

#### scDNA relaxation assays: TOP2A enzyme

Two units of the TOP2A enzyme were incubated with 200 ng of scDNA in reaction buffer A [containing 50 mM tris-HCl (pH 8.0), 150 mM NaCl, 10 mM MgCl_2_, 0.5 mM DTT, and bovine serum albumin (30 μg/ml)], 2 mM ATP in the presence of etoposide as a control inhibitor, and LZD, PNU142586, or PNU142300 to a final volume of 20 μl. The reaction was conducted on ice and then incubated at 37°C for 30 min before being stopped by adding 2 μl of 10% SDS, followed by addition of 2 μl of 10× gel loading buffer (bromophenol and glycerol). The reaction was resolved using electrophoresis on 1% agarose gel at 135 V for 30 min. After staining with RedSafe in 1× tris-acetate-EDTA buffer for 30 min, the DNA bands were visualized using the ChemiDoc Imaging System (Bio-Rad).

#### scDNA relaxation assays: Top2B enzyme

One unit of the TOP2B enzyme was incubated with 200 ng of scDNA in a reaction assay buffer [5 mM tris-HCl (pH 7.5), 12.5 mM NaCl, 1 mM MgCl_2_, 0.5 mM DTT, and albumin (10 μg/ml)], 0.1 mM ATP in the presence of etoposide as a control inhibitor, and LZD, PNU142586, or PNU142300 to a final volume of 30 μl. The reaction was conducted on ice and then incubated at 37°C for 30 min before being stopped by adding 2 μl of 10% SDS, followed by addition of 2 μl of 10× gel loading buffer (bromophenol and glycerol). The reaction was resolved by electrophoresis on a 1% agarose gel at 135 V for 30 min. After staining with RedSafe in 1× tris-borate EDTA buffer for 30 min, the DNA bands were visualized using the ChemiDoc Imaging System (Bio-Rad).

#### DNA cleavage assays

The complete 10× reaction assay buffer was combined between buffer A (no ATP) and buffer B (ATP) at a ratio of 1:1. Following this, three units of the TOP2A enzyme and two units of the TOP2B enzyme were incubated with 200 ng of scDNA in 1× reaction assay buffer [containing 5 mM tris-HCl (pH 7.5), 12.5 mM NaCl, 1 mM MgCl_2_, 0.5 mM DTT, and albumin (10 μg/ml)], 2 mM ATP in the presence of VP16/etoposide, or DMSO or PNU142586 to a final volume of 20 μl. Cleavage VP16 replacement was conducted using TOP2A and TOP2B enzymes incubated with scDNA in the presence of VP16 and novobiocin or PNU142586 in 1× reaction buffer with 2 mM ATP. The reaction was incubated at 37°C for 30 min and then stopped by adding 2 μl of 10% SDS and 1 μl of 0.5 M EDTA before digestion with 2 μl of protease K (50 μg/ml). The reaction was followed by incubation at 45°C for 30 min and then the addition of 2 μl of 10× gel loading buffer (bromophenol and glycerol). The reaction was resolved using electrophoresis with two different 1% agarose gels (with and without ethidium bromide in tris-acetate-EDTA buffer) at 135 V for 30 min. The DNA bands were visualized using the ChemiDoc Imaging System (Bio-Rad).

### Ethidium bromide displacement assays

Ethidium bromide displacement assays were conducted as previously described (*56*, *112*, *113*) using m-AMSA as a positive control. In a reaction volume of 20 μl, PNU142586 was serially diluted from 2.5 mM to 4 μM (1.48 mg/liter) and mixed with salmon sperm DNA (100 μg/ml) and ethidium bromide (2 μg/ml) in fluorescence buffer containing 10 mM Hepes (pH 7.9), 100 mM KCl, 5 mM MgCl_2_, and 0.1 mM EDTA. The reactions were conducted in triplicate and subjected to fluorescence emissions of 590 nm and excitation of 535 nm. Signals were detected using a SpectraMax iD3 Multimode Microplate Reader.

### Expression and purification of the recombinant ATPase and DNA binding domains of TOP2A

For the ATPase domain, the DNA sequences of N-terminal 6× His and the maltose binding protein (MBP) tag flanked by residues 29 to 428 in human TOP2A were synthesized. The MBP-encoding sequence was referenced to pIADL 16, and tobacco etch virus protease recognition sequences were inserted between the MBP tag and TOP2A. The synthesized His-MBP–tagged TOP2A ATPase domain was cloned in pET24a (Novagen) with Nde I and Xho I. BL21(DE3) (Inbionet) was transformed with the cloned plasmid. Cultures were grown in LB media at 37°C. When an absorbance at 600 nm of 0.6 to 0.8 was achieved, isopropyl 1-thio-β-d-galactopyranoside (GoldBio) was added to a final concentration of 0.5 mM before incubation at 25°C. The cells were harvested with centrifugation and lysed using sonication. The His-MBP–tagged TOP2A ATPase domain was purified in an Ni-NTA column (Roche) and cleaved with tobacco etch virus protease to produce His-MBP and the TOP2A ATPase domain. The imidazole of the cleaved product was removed in a desalting column (Cytiva) and run through the Ni-NTA column again to eliminate His-MBP. The TOP2A ATPase domain was purified further using a heparin column (Cytiva).

For the DNA binding domain, the DNA sequences of residues 429 to 1188 in human TOP2A flanked by the C-terminal 10× His tag were synthesized. The synthesized C-terminal His-tagged TOP2A DNA binding domain was cloned in pET24a (Novagen) with Bg III and Xho I. BL21(DE3) (Inbionet) was transformed with the cloned plasmid. Cultures were grown in LB media at 37°C. When an absorbance at 600 nm of 0.6 to 0.8 was achieved, isopropyl 1-thio-β-d-galactopyranoside (GoldBio) was added to a final concentration of 0.5 mM, followed by incubation at 17°C. The cells were harvested with centrifugation and lysed using sonication. The C-terminal His-tagged TOP2A DNA binding domain was enriched in the Ni-NTA column (Roche) and further purified in a heparin column (Cytiva).

### TFQ assays

All TFQ experiments were conducted using a SpectraMax iD3 Multimode Microplate Reader. The TOP2A enzyme was prepared by diluting it with a reaction buffer [containing 20 mM tris-HCl (pH 7.5), 50 mM NaCl, and 2 mM DTT] to reach final concentrations of 3.83 and 3.00 μM for the ATPase domain and DNA binding domain, respectively. It was then mixed in a microplate with LZD, PNU142300, and PNU142586 to final concentrations of 500, 250, 125, 62.5, and 31.25 μM. Each reaction (50 μl each) was transferred to a half-area UV-star 96 well microplate. Trp fluorescence was selectively measured at 25°C with excitation at 280 nm over an emission wavelength range of 320 to 500 nm. For competition TFQ assays, the results were compared with and without the CTD linker peptide (residues 1193 to 1217). The maximum Trp fluorescence of the TOP2A DNA binding domain titrated with PNU142586 in the presence of the CTD linker peptide exhibited a lower fluorescence intensity across all titrated concentrations of PNU142586 compared to the absence of the linker peptide. Fluorescence titration was conducted with LZD, PNU142300, and PNU142586 at a single wavelength, and the results were normalized by dividing the measured fluorescence (*F*) by the fluorescence measured in the absence of the compound (*F*_o_) for the TOP2A DNA binding domain or ATPase domain. The relationship between *F*/*F*_o_ and the ligand concentrations for each TFQ experiment was analyzed using nonlinear regression with a site-specific binding model, *A* = *B*_max_ × *C*/(*K*_D_ + *C*), where *C* is the ligand concentration, *B*_max_ is the maximum specific binding, and *K*_D_ is the equilibrium binding constant, using R (https://cran.r-project.org/).

### Electrophoresis mobility shift assays (EMSAs)

The EMSAs were conducted following the standard protocols with minor modifications. The single-strand DNA oligo 5ZRF (AGCCGAGCTGCAGCTCGG CT) was labeled using a Pierce Biotin 3′ End DNA Labeling Kit (Thermo Fisher Scientific, Pierce) and thermo PCR. Following this, 20 μM human TOP2A-DNA binding site domain was incubated with 300 nM 5ZRF oligo in a standard 20-μl EMSA reaction in a reaction buffer containing 20 mM Hepes, 2 mM DTT, 3 mM MgCl_2_, 0.05% NP-40, and 0.1 μg of poly[dI:dC]. After incubation for 30 min at room temperature, the reactions were analyzed using electrophoresis on 7.5% nondenaturing polyacrylamide gel at 100 V. Thirty minutes after transfer, the membrane was cross-linked using ultraviolet light for 5 min and the DNA oligo bands were visualized using chemiluminescence (Thermo Fisher Scientific, Pierce).

### Cell-based assays

#### Proliferation assays

Cell proliferation was measured using CellTiter 96 AQueous nonradioactive cell proliferation assays (Promega, Madison, WI) according to the manufacturer’s instructions. After incubation with the reagents, the cells were directly treated with MTS tetrazolium salt for 2 hours at 37°C. The absorbance was then read using a SpectraMax iD3 machine at 490 nm.

#### Cytotoxicity assays

Cytotoxicity was evaluated using commercial kit CyQUANT LDH cytotoxicity assays (Invitrogen). The culture media were collected after centrifugation and directly used to measure lactate dehydrogenase levels using a colorimetric method following the manufacturer’s protocol. After 30 min of incubation, the absorbance was measured using a SpectraMax iD3 machine at 490 nm.

#### RNA interference

HL-60 cells grown to more than 90% viability were incubated in RPMI-1640 containing 10% fetal bovine serum and an antibiotic. siRNAs for TOP2A, TOP2B, or a universal scrambled negative control siRNA duplex were transfected into the cells using HiPerFect (Qiagen) according to the manufacturer’s protocols. The siRNA oligonucleotides and HiPerfect were mixed in fresh media with a final concentration of 10 nM in each well. After 10 min of incubation, the mixture was dropped onto the cells and maintained for 6 hours. Complete culture media were supplied to the cells, which were collected after 48 hours.

#### Apoptosis analysis

The HL-60 cells were grown in six-well plates and treated with reagents for 48 hours. The cells were then collected and washed twice with cold phosphate-buffered saline and resuspended in 1× binding buffer at a concentration of 1 × 10^6^ cells/ml. Following this, 100 μl of each solution was transferred to a tube and then stained with 5 μl of fluorescein isothiocyanate annexin V and 5 μl of propidium iodide for 15 min at room temperature in the dark. The samples were then analyzed using BD Bioscience FACSVerse, and the results were visualized with FlowJo version 10 software.

#### Western blot analysis

Cells were treated and were lysed in radioimmunoprecipitation assay buffer containing protease and a phosphatase inhibitor cocktail tablet. Cell lysates were separated on polyacrylamide gel after being boiled for 5 min at 95°C and then wet transferred to polyvinylidene difluoride membranes. The membranes were blocked in skim milk before being incubated with the indicated primary antibodies against β-actin (A1978, Sigma-Aldrich), MT-CO1 (Invitrogen), Topo IIα (Abcam), Topo IIβ (BD Transduction Laboratories), cleaved PARP (Cell Signaling Technology), and phosphorylated H2AX (Merck). The membranes were then incubated with horseradish peroxidase–conjugated secondary antibodies and visualized using a ChemiDoc Imaging System (Bio-Rad) after incubation with a Clarity Western ECL substrate.

#### RNA extraction and RT-qPCR

RNA from THP-1 and HL-60 was extracted following the instructions for the TRIzol reagent (Invitrogen). cDNA was then synthesized using a Maxima First Strand cDNA Synthesis Kit for RT-qPCR (Thermo Fisher Scientific), and RT-qPCR was conducted using GoTaq qPCR Master Mix (Promega). The samples were amplified using the primer sets listed in Table 1. The human *GAPDH* (glyceraldehyde-3-phosphate dehydrogenase) gene was used as an endogenous control for sample normalization. Using the 2^−ΔΔCt^ method, the relative abundance of the target mRNA for each sample was determined on the basis of the −Δcycle threshold (Δ*C*_t_) values of the target and endogenous *GAPDH* reference genes.

### In vivo functional assays

#### Ethics statement for animal experiments

All animal experiments were approved by Inje University Busan Paik Hospital Institutional Animal Care and Use Committee (IACUC, Busan, South Korea) in accordance with the Animal Protection Act (IJUBPH_2023-010-02).

#### Xenopus laevis *model*

Wild-type *Xenopus laevis* females were obtained from the Korea Xenopus Resource Center for Research. All of the experiment procedures were conducted at 18°C. *X. laevis* individuals were anesthetized in an anesthetic solution (0.1% 3-aminobenzoic acid ethyl ester and 0.3% potassium bicarbonate; Sigma-Aldrich). Oocytes were surgically obtained and washed in ORII buffer [82.5 mM NaCl, 2 mM KCl, 1 mM MgCl_2_·6H_2_O, and 5 mM Hepes (pH 7.4)] (*114*). The extracted oocytes were defolliculated in an enzyme solution (collagenase A: 20 mg/15 ml of 1× ORII buffer) and kept in Barth’s solution [88 mM NaCl, 1 mM KCl, 0.41 mM CaCl_2_·2H_2_O, 0.33 mM Ca(NO_3_)_2_·4H_2_O, 0.82 mM MgSO_4_·7H_2_O, 2.4 mM NaHCO_3_, and 5 mM Hepes (pH 7.4)] in an incubator at 18°C overnight (*115*).

#### Zebrafish model

Wild-type zebrafish lines were obtained from the Zebrafish Center for Disease Modeling (ZCDM), Chungnam National University, South Korea. Wild-type adult zebrafish were maintained and bred under a 14-hour light/10-hour dark cycle (*116*) at 28°C and fed with GEMMA Micro 300. Three healthy male/female pairs of adult zebrafish were kept separately in a mating cage in the afternoon the day before mating to obtain embryos. Embryos were collected within the first hour postfertilization and raised in egg water [Instant Ocean Sea Salt (60 μg/ml)] at 28°C.

#### Xenopus *oocyte extract decatenation assays*

*Xenopus* oocyte extracts were prepared from female *X. laevis*, as previously described with modifications (*117*, *118*). Pooled oocytes in Barth’s solution were washed three times with lysis buffer [50 mM Hepes (pH 7.9), 50 mM KCl, 5 mM MgCl_2_, and 5 mM DTT]. The oocytes were then allowed to settle using centrifugation at 150*g* for 1 min, and the supernatant was discarded. The oocytes were then homogenized (40 manual strokes with a plastic pestle) with lysis buffer supplemented with aprotinin (10 μg/ml), pepstatin A (10 μg/ml), and leupeptin (10 μg/ml) and centrifuged (20,000*g* for 15 min) at 2°C. Following centrifugation, the lipids were aspirated and the soluble supernatant was recovered, frozen in liquid nitrogen, and then stored at −80°C.

After thawing the frozen *Xenopus* oocyte extract, it was incubated with 200 ng of kDNA in reaction assay buffer [containing 5 mM tris-HCl (pH 7.5), 12.5 mM NaCl, 1 mM MgCl_2_, 0.5 mM DTT, and albumin (10 μg/ml)], 0.5 mM ATP in the presence of novobiocin as a control inhibitor, and LZD or PNU142586 to a final volume of 30 μl. The reaction was conducted on ice and then incubated at 37°C for 30 min before being stopped by adding 5 μl of 5× stop buffer (5% Sarkosyl, 0.125% bromophenol blue, and 25% glycerol). The reaction was digested with ribonuclease A (Thermo Fisher Scientific) and proteinase K (Inspiralis) and was then resolved using electrophoresis on 1% agarose gel with RedSafe at 135 V for 30 min. DNA bands were visualized using the ChemiDoc Imaging System (Bio-Rad).

#### *Toxicological studies using* X. laevis *oocytes*

The extracted oocytes with a clear distinction between brown and white in the V-VI stages were selected under a microscope and randomly transferred to a 48-well plate with 10 oocytes per well. Each well was treated with 300 μl of the test solution at different concentrations, including the control (2% DMSO). The treatment plate was covered with aluminum foil and incubated at 18°C for 7 days. After the 7-day incubation period, the morphological changes to treated oocytes were observed and evaluated under a microscope.

#### Toxicological studies using zebrafish larvae

At 24 hpf, well-developed embryos were manually dechorionated using forceps and transferred to a 24-well plate with 10 embryos per well. Each well was given 1 ml of the test compound in egg water at different concentrations, including a control (0.1% DMSO). The treatment plate was incubated in a 28°C incubator. The morphology and mortality of the zebrafish embryos were observed daily under the microscope until 120 hpf (*119*). Larvae were anesthetized using tricaine (Sigma-Aldrich) and immobilized on 3% methylcellulose gel (Sigma-Aldrich). The images were obtained using a TOMLOV digital microscope.

#### Gene expression analysis using qPCR in zebrafish

At 120 hpf, treated zebrafish larvae from each well were collected after imaging. Total RNA was extracted using the TRIzol reagent (Invitrogen), and cDNA was synthesized using a Maxima First Strand cDNA Synthesis Kit (Thermo Fisher Scientific). RT-qPCR was conducted with the cDNA samples using GoTaq qPCR Master Mix (Promega).

#### Quantification and statistical analysis

Unless otherwise specified, all experiment results were conducted in triplicate and data are presented as the means ± SD or means ± SEM, as described in the figure legends. Statistical significance was analyzed using GraphPad Prism (version 8.0.2, GraphPad Software Inc., US). A *P* value of ≤ 0.05 was considered a significant difference using *t* test analysis.

### Figure preparation

Figures were produced using GraphPad Prism 8.0.2 (GraphPad Software), R version 4.3.2 (https://cran.r-project.org/), and PyMOL (www.pymol.org).
