# Supplementary material for: Deciphering linezolid-induced hematologic toxicity: Targeting TOP2A and TOP2B via its primary metabolite PNU142586
Source: Sci Adv. 2025 May 28;11(22):eadt5833. doi: 10.1126/sciadv.adt5833 (PMC12118551; doi:10.1126/sciadv.adt5833)
Supplement: Supplementary file 1 — Figs. S1 to S11 Tables S1 to S6 Legend for data S1 [file sciadv.adt5833_sm.pdf]

Science Advances
AAAS

Supplementary Materials for

Deciphering linezolid-induced hematologic toxicity: Targeting TOP2A and TOP2B via its primary metabolite PNU142586

Vo Thuy Anh Thu *et al.*

Corresponding author: Yong-Soon Cho, ysncho@gmail.com

Sci. Adv. **11**, eadt5833 (2025)
DOI: 10.1126/sciadv.adt5833

The PDF file includes:

Figs. S1 to S11
Tables S1 to S6
Legend for data S1

Other Supplementary Material for this manuscript includes the following:

Data S1

Supplementary Materials

Figures

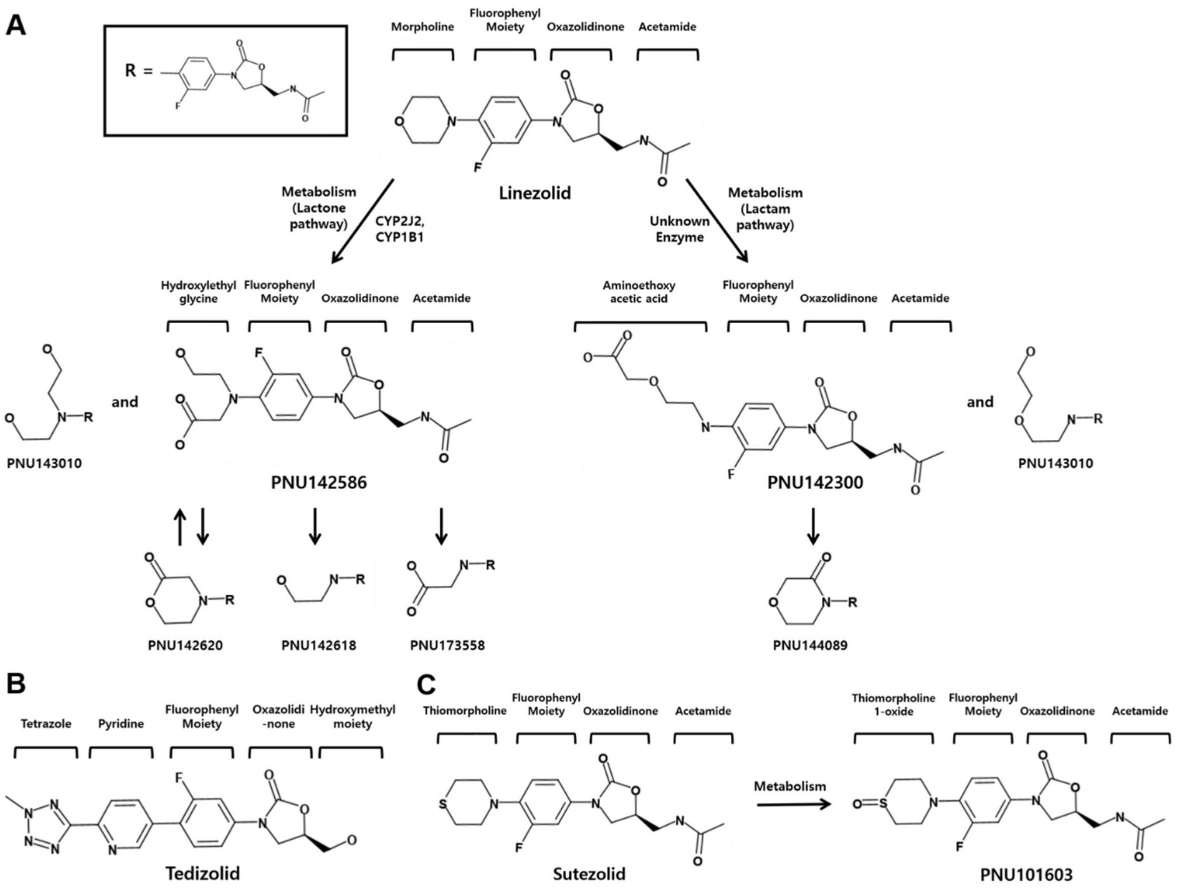

Fig. S1. Molecular structures and metabolic pathway of linezolid, tedizolid, sutezolid, and their major metabolites. The figure illustrates the structure and metabolic pathway of linezolid, including its major metabolites, PNU142586 and PNU142300, as well as other minor metabolites (A). Additionally, it presents the structures of tedizolid (B) and sutezolid along with its metabolite, PNU101603 (C). Key functional groups are labeled in each structure for clarity.

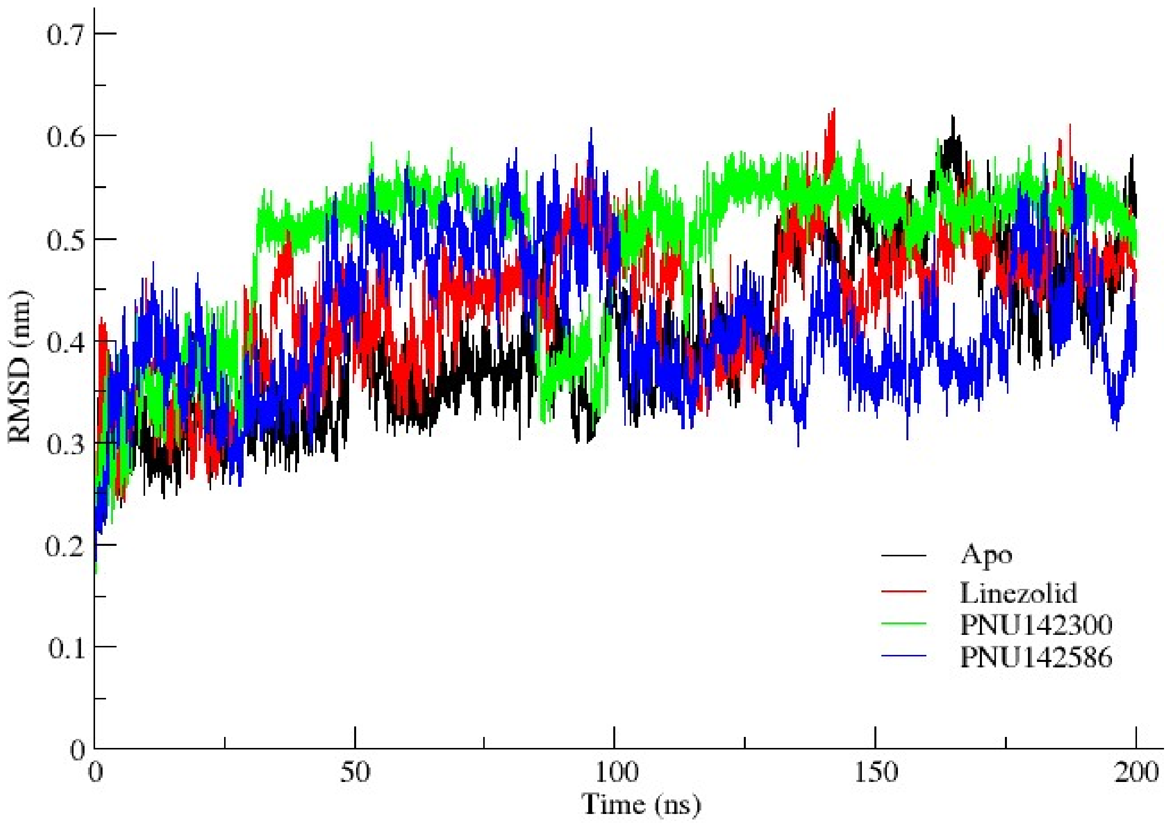

Fig. S2. Molecular dynamics of the TOP2A ATPase domain. C-α root mean square deviation (RMSD) of the TOP2A ATPase domain in the absence of ligands (Apo, black) and when docked with linezolid (red), PNU142300 (green), and PNU142586 (blue) during the molecular dynamics simulations.

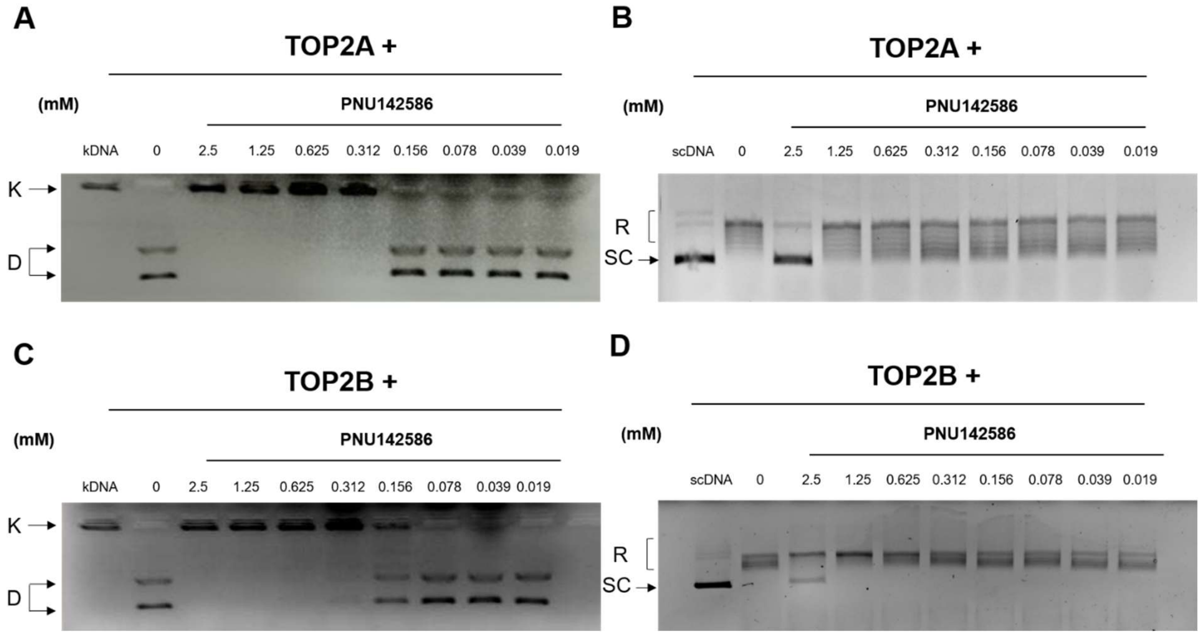

Fig. S3. IC_{50} measurement of PNU142586 activity against the decatenation and relaxation activity of TOP2A and TOP2B. Kinetoplast DNA (kDNA) decatenation assays were used to determine the TOP2A **(A)** and TOP2B **(C)** activity inhibited by PNU142586 (mM). The decatenation assays were repeated three times for each concentration. Supercoil DNA (scDNA) relaxation assays were used to determine TOP2A **(B)** and TOP2B **(D)** activity inhibited by PNU142586 (mM). The relaxation assays were repeated three times. K, catenated kDNA; D, decatenated kDNA; SC, supercoiled forms of the plasmid; R, relaxed forms of the plasmid; TOP2A, DNA topoisomerase 2-alpha; TOP2B, DNA topoisomerase 2-beta.

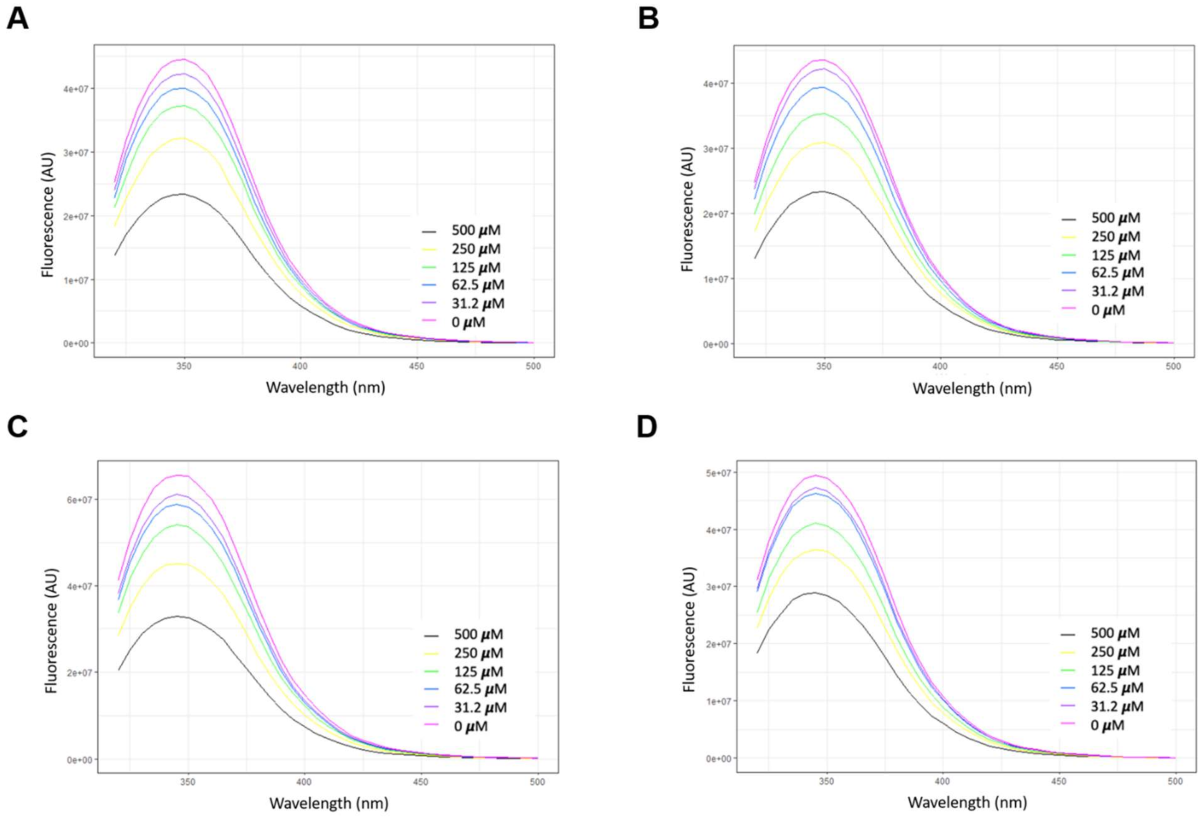

Fig. S4. Titration of TOP2A with linezolid and PNU142300 Tryptophan (Trp) fluorescence spectra for the TOP2A DNA binding domain **(A)** and ATPase domain **(C)** with higher concentrations of linezolid were recorded using fluorescence spectroscopy. The inserts in each panel show the titrated concentration of linezolid. Trp fluorescence spectra for the TOP2A DNA binding domain **(B)** and ATPase domain **(D)** with higher concentrations of PNU142300 were recorded Using fluorescence spectroscopy. The inserts in each panel show the titrated concentration of PNU142300. AU indicates an arbitrary unit. TOP2A; DNA topoisomerase 2-alpha.

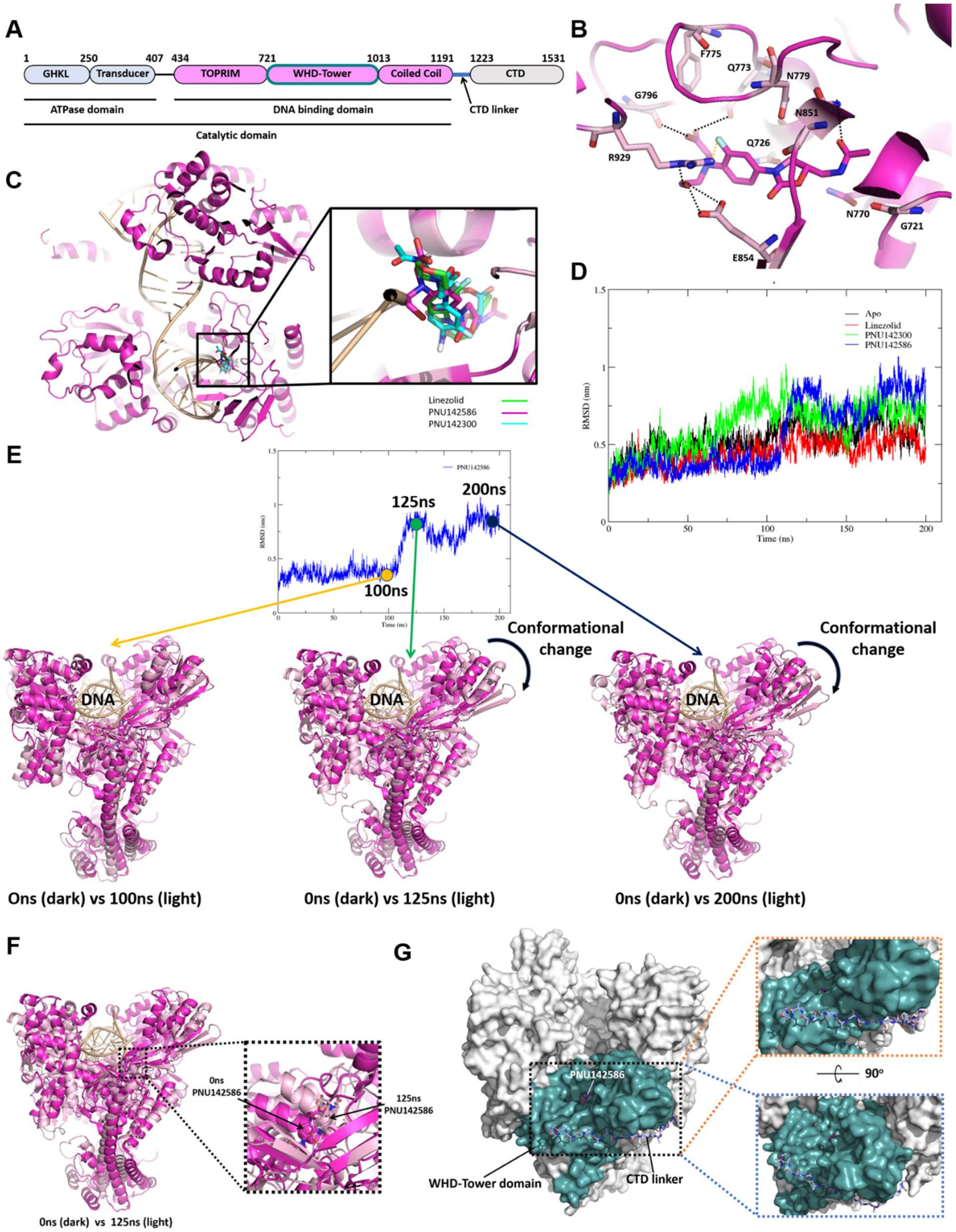
A
1
250
407
434
721
1013
1191
1223
1531
GHKL
Transducer
TOPRIM
WHD-Tower
Coiled Coil
CTD
ATPase domain
DNA binding domain
CTD linker
Catalytic domain
B
F775
Q773
N779
G796
N851
Q726
R929
N770
G721
E854
C
Linezolid
PNU142586
PNU142300
D
Apo
Linezolid
PNU142300
PNU142586
RMSD (nm)
Time (ns)
E
PNU142586
125ns
200ns
100ns
Conformational change
DNA
0ns (dark) vs 100ns (light)
0ns (dark) vs 125ns (light)
0ns (dark) vs 200ns (light)
F
0ns PNU142586
125ns PNU142586
0ns (dark) vs 125ns (light)
G
PNU142586
90°
WHD-Tower domain
CTD linker

Fig. S5. Putative binding mode of PNU142586 with the TOP2A DNA binding domain. (A) Schematic domain organization of TOP2A. **(B)** Close-up view of the putative binding pocket showing interactions between the DNA-binding domain and PNU142586. Interacting residues are shown as sticks, and hydrogen bonds as black dotted lines. Hydrogen atoms are omitted for clarity. **(C)** Substrate DNA segments of TOP2A are superimposed onto the DNA-binding domain docked with linezolid (green), PNU142586 (magenta), and PNU142300 (cyan). The binding pocket is shown in a close-up view. Proteins and DNA are depicted as magenta and yellow cartoon models, respectively. **(D)** C-α RMSD of the DNA-binding domain is plotted over time for the apo form (black) and complexes with linezolid (red), PNU142586 (blue), and PNU142300 (green) during molecular dynamics simulations. **(E)** RMSD plot (upper) of the DNA-binding domain bound to PNU142586 over 200 ns. Structures at 100, 125, and 200 ns (light magenta) are overlaid with the initial structure (dark magenta, lower panel). DNA is shown as a yellow cartoon model. **(F)** Superimposed structures of the domain with PNU142586 at 0 ns (dark magenta) and 125 ns (light magenta) show conformational changes. Inset: close-up of the stable binding of PNU142586 within the pocket despite structural rearrangement. **(G)** The CTD linker (light blue) from the full-length cryo-EM structure (PDB ID: 6zy8) is aligned to the DNA-binding domain (PDB ID: 5gwk) docked with PNU142586 (magenta). The WHD-Tower domain is highlighted in dark green. The drug-binding and allosteric sites are boxed (black) and magnified (orange: parallel orientation; blue dashed: orthogonal orientation). Protein surfaces are shown in the magnified views. TOP2A, DNA topoisomerase 2-alpha.

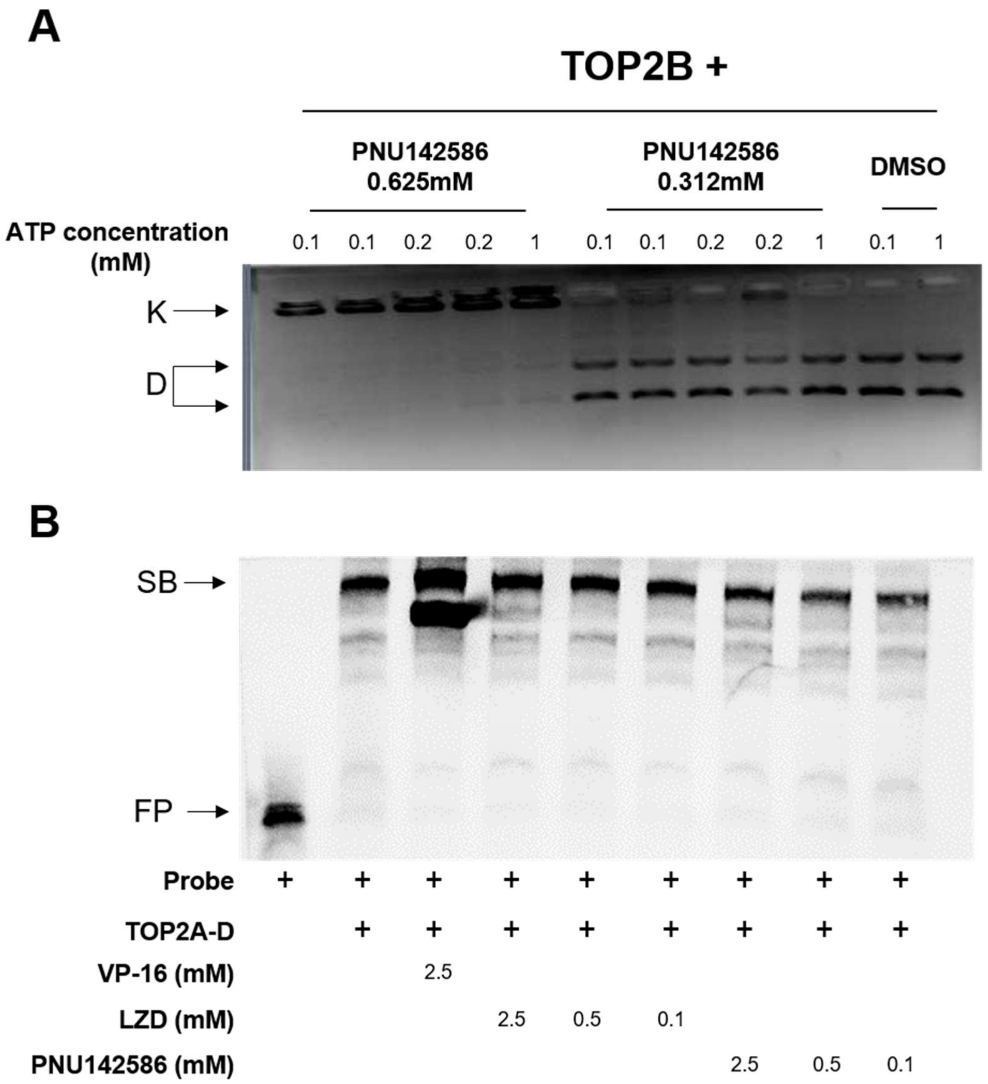

Fig. S6. PNU142586 exhibits a dual molecular mode of action in inhibiting TOP2 (A) Kinetoplast DNA (kDNA) decatenation assays measured the TOP2B activity inhibited by PNU142586 with the increasing concentrations of ATP (mM). K, catenated kDNA; D, decatenated kDNA; TOP2B, DNA topoisomerase 2-beta. EMSAs were performed by incubating 20 μM purified human TOP2A-D with 300 nM Biotin-labeled 5ZRF DNA oligo. Following this, 0.5–2.5 mM etoposide (VP-16), linezolid, or PNU142586 was added to the reaction as shown in **(B)**. SB, Shifted band; FP, Free probe; TOP2A-D, Topoisomerase 2-alpha DNA binding site.

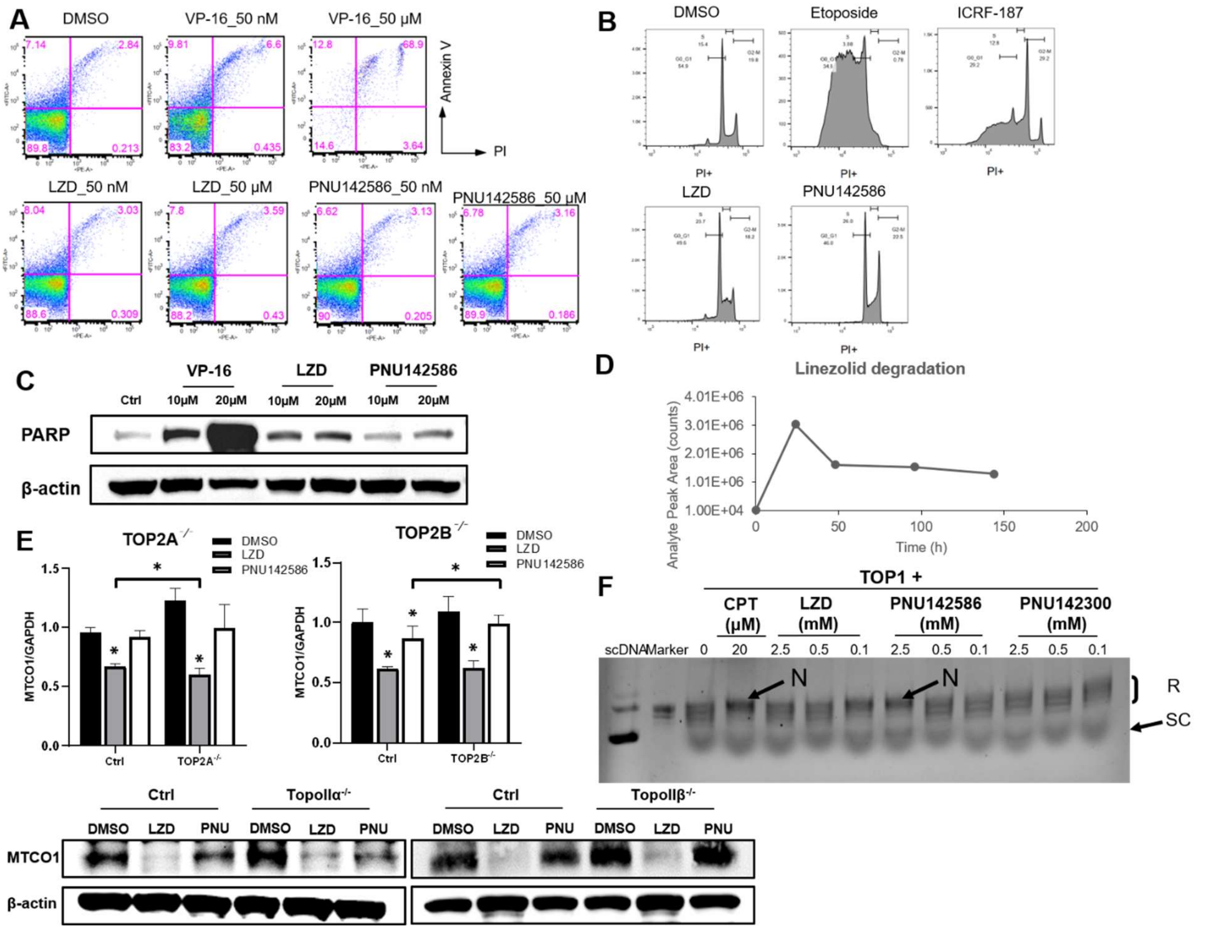

Fig. S7. PNU142586 inhibit proliferation and MT-CO1 in human blood cell lines (**A**) Annexin V and PI staining in HL-60 cells treated with 50 nM or 50 µM of etoposide (VP-16), linezolid or PNU142586 with DMSO treatment as a control after incubation for 48 h. The cells were analyzed using BD Bioscience FACSVerse and the results were visualized by with FlowJo™ v10 software. (**B**) HL-60 cells treated with DMSO (control), Linezolid (LZD), PNU142586, Etoposide, or ICRF-187 at a concentration of 50 µM for 48 hours. Flow cytometry was performed using the BD Cycletest™ Plus DNA Reagent Kit to stain isolated nuclei, enabling the estimation of cell cycle phase distributions (G1, S, and G2/M). **(C)** HL-60 cells were treated with DMSO or 10–20 µM etoposide (VP-16), 10–20 µM linezolid or PNU142586 for 48 h. Immunoblotting measured

cleaved PARP levels in cells with β-actin as a loading control. (**D**) HL-60 cells were incubated with 50 μM of Linezolid for various time points (0 h, 24 h, 48 h, 96 h, and 144 h). After incubation, the cells were washed with ice-cold PBS, and lysates were prepared for LC-MS/MS analysis by adding 100 μl of 70% acetonitrile (ACN), followed by sonication and centrifugation. The resulting lysates were analyzed using LC-MS/MS to quantify Linezolid levels. (**E**) Cells were knocked down TOP2A and TOP2B using siRNA. After 72 h, the media was changed, and cells were treated with linezolid or PNU142586. RNA was then extracted for gene expression analysis or MTCO1 levels were detected for Western blots. * $p < 0.05$ vs. DMSO. (**F**) Supercoil DNA (scDNA) relaxation activity of TOP1 was measured in the presence of linezolid or its metabolites PNU142586 and PNU142300 (mM). Camptothecin (CPT) was used as the positive control. SC, supercoiled forms of the plasmid; R, relaxed forms of the plasmid; Marker, relaxed marker; TOP1, DNA topoisomerase 1

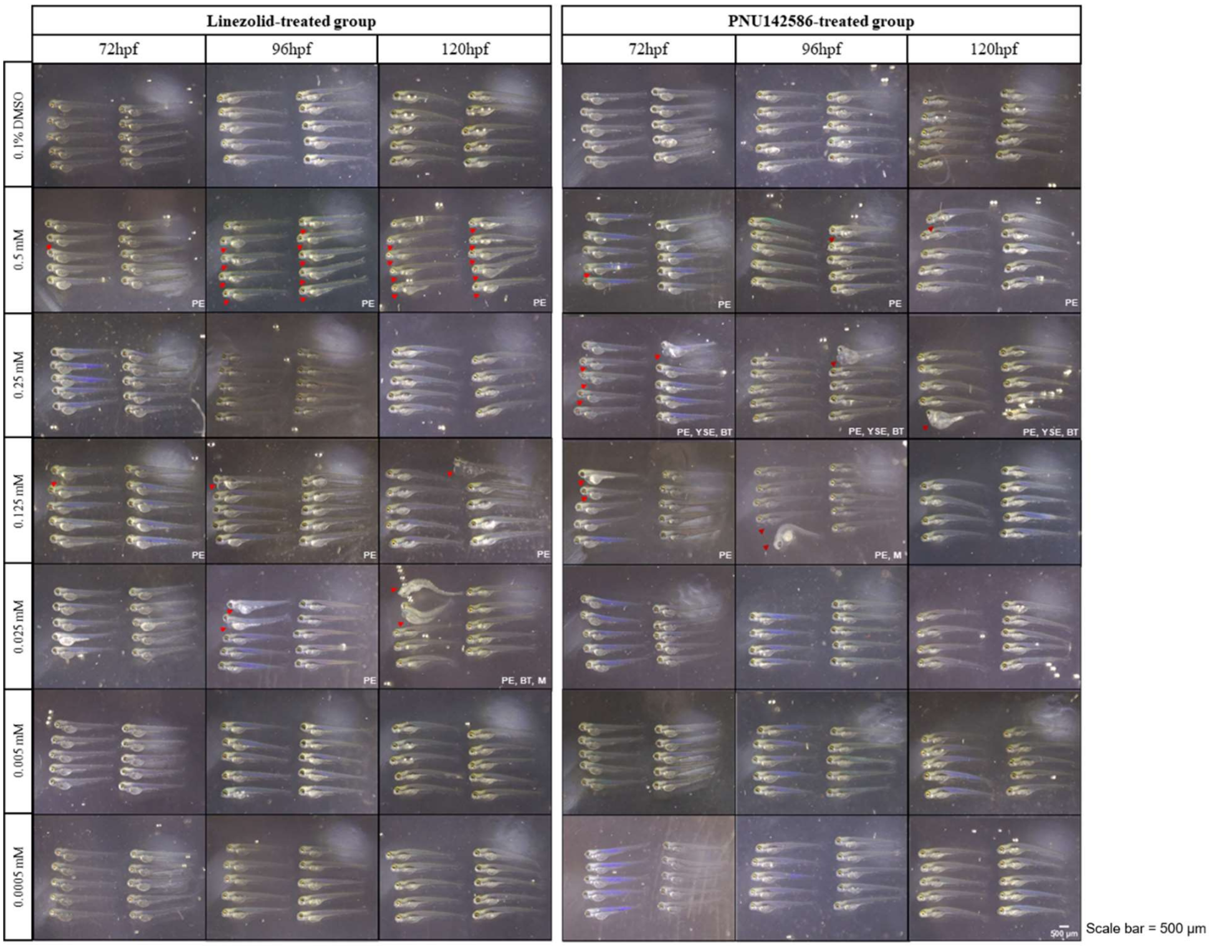

Fig. S8. Effects of Linezolid and PNU142586 on the development of zebrafish larvae. Well-developed 24 hpf embryos were manually dechorionated and exposed to linezolid and PNU142586. The morphology was observed daily and images recorded until 120 hpf. Examples of malformation are indicated by the red arrows. M, mortality; PE, pericardial edema, YSE, yolk sac edema; BT, bent tail. Scale bar = 500 μm (*n*=30; 10 larvae in triplicate for each group).

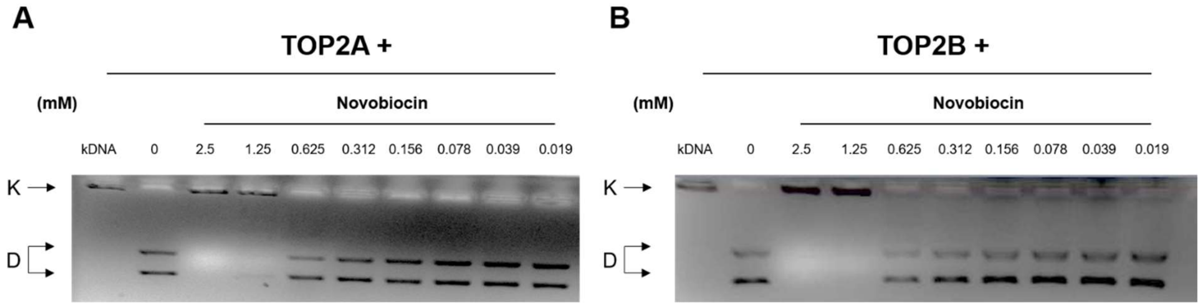

Fig. S9. IC_{50} measurement of Novobiocin activity against the decatenation activity of TOP2A and TOP2B. Kinetoplast DNA (kDNA) decatenation assays were used to determine the TOP2A (**A**) and TOP2B (**B**) activity inhibited by Novobiocin (mM). K, catenated kDNA; D, decatenated kDNA; TOP2A, DNA topoisomerase 2-alpha; TOP2B, DNA topoisomerase 2-beta.

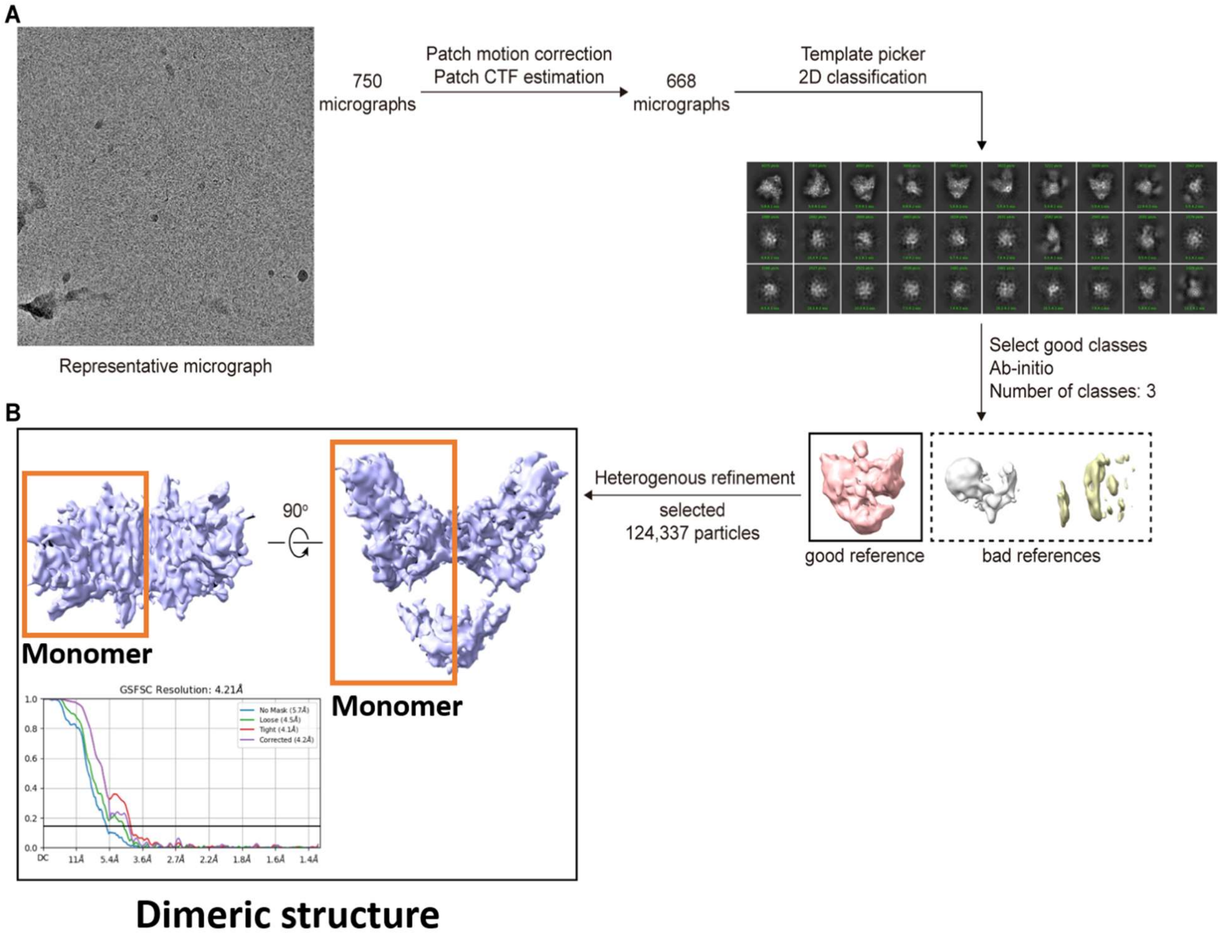

Fig. S10. Cryo-EM data analysis of the recombinant DNA-binding domain of TOP2. (A) Overall workflow of the cryo-EM image analysis. Motion correction and CTF estimation were performed using cryoSPARC V3.1.4. 82 images with poor statistics were discarded. Particles were picked using template picker and further classified by subsequent 2D classification. 2D classes with moderate features of the TOP2A and good resolutions are selected and subjected to generate reference model via ab-initio. Subsequently, reference-based 3D classification was conducted using three input models and 124,337 particles in the best class were selected. NU-refinement was performed to generate a high-resolution map. **(B)** Cryo-EM structure of the TOP2A DNA binding

domain. All resolutions are estimated using the gold-standard Fourier shell correlation 0.143 criteria.

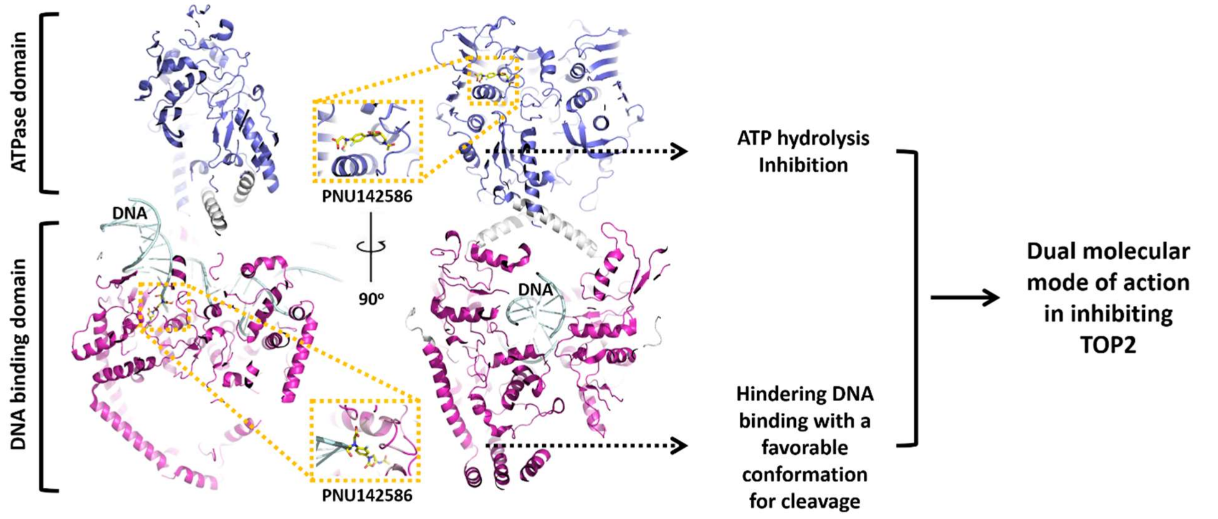

Fig.S11. Dual mechanism of action of PNU142586 in inhibiting TOP2. The proposed dual molecular mechanism of action of PNU142586 as a catalytic inhibitor of TOP2 is illustrated. Using the full-length cryo-EM structure of TOP2 (PDB ID 6ZY7), the ATPase domain, DNA-binding domain, and DNA are shown in blue, magenta, and cyan, respectively. The two primary inhibitory actions of PNU142586 are highlighted, with insets providing close-up views of its interactions with these domains. The structure is also rotated by 90° to offer an alternative perspective, further clarifying the spatial relationships between the domains and the compound's binding interactions.

Table S1 Potential human protein targets of linezolid and linezolid metabolites PNU142586 and PNU142300 predicted using ACID.

Compound	Target Rank Criteria	Rank	PDB ID	Protein Name
Linezolid	ΔE bind[a]	1	1AUT	Vitamin K-dependent protein C
		2	1EXA	Retinoic acid receptor gamma
		3	1XAP	Retinoic acid receptor beta
		4	7EIW	Histidine decarboxylase
		5	2HI4	Cytochrome P450 1A2
		6	3ISQ	4-hydroxyphenylpyruvate dioxygenase
		7	1H9U	Retinoic acid receptor RXR-beta
		8	1O6U	SEC14-like protein 2
		9	1G13	Ganglioside GM2 activator
		10	1BJ1	Vascular endothelial growth factor A, long form
	Docking score	1	3A7E	Catechol O-methyltransferase
		2	1GW6	Leukotriene A-4 hydrolase
		3	3ORH	Guanidinoacetate N-methyltransferase
		4	2BZG	Thiopurine S-methyltransferase
		5	6PVS	Nicotinamide N-methyltransferase
		6	3LMK	Metabotropic glutamate receptor 5
		7	2CKJ	Xanthine dehydrogenase/oxidase
		8	1GOS	Monoamine oxidase B
		9	1AUT	Vitamin K-dependent protein C
		10	2W8N	Succinate-semialdehyde dehydrogenase, mitochondrial
PNU142586	ΔE bind[a]	1	3II0	Aspartate aminotransferase, cytoplasmic

		2	3ZCF	Cytochrome C
		3	1XWW	Low molecular weight phosphotyrosine protein phosphatase
		4	3L6B	Serine racemase
		5	1M73	Purine nucleoside phosphorylase
		6	1A8E	Serotransferrin
		7	3RBF	Aromatic-L-amino-acid decarboxylase
		8	1NB0	Riboflavin kinase
		9	2BZG	Thiopurine S-methyltransferase
		10	3B6R	Creatine kinase B-type
	Docking score	1	3II0	Aspartate aminotransferase, cytoplasmic
		2	2I3C	Aspartoacylase
		3	2BZG	Thiopurine S-methyltransferase
		4	1GW6	Leukotriene A-4 hydrolase
		5	3C6K	Spermine synthase
		6	6PVS	Nicotinamide N-methyltransferase
		7	4F11	Gamma-aminobutyric acid type B receptor subunit 2
		8	2CKJ	Xanthine dehydrogenase/oxidase
		9	1M73	Purine nucleoside phosphorylase
		10	1GOS	Monoamine oxidase B
PNU142300	ΔE bind[a]	1	2NMP	Cystathionine gamma-lyase
		2	3L6B	Serine racemase
		3	7EIW	Histidine decarboxylase
		4	1ZXM	DNA topoisomerase 2-alpha
		5	1EFH	Sulfotransferase 2A1

		6	1FW1	Maleylacetoacetate isomerase
		7	1ORE	Adenine phosphoribosyltransferase
		8	1PDG	Platelet-derived growth factor subunit B
		9	1WMS	Ras-related protein Rab-9A
		10	1TQN	Cytochrome P450 3A4
	Docking score	1	2NMP	Cystathionine gamma-lyase
		2	6PVS	Nicotinamide N-methyltransferase
		3	1GW6	Leukotriene A-4 hydrolase
		4	4F11	Gamma-aminobutyric acid type B receptor subunit 2
		5	2BZG	Thiopurine S-methyltransferase
		6	1DKF	Retinoic acid receptor alpha
		7	3ORH	Guanidinoacetate N-methyltransferase
		8	3C6K	Spermine synthase
		9	2CKJ	Xanthine dehydrogenase/oxidase
		10	5Z62	Cytochrome c oxidase subunit 2

a ΔE bind: the change in the binding energy

Table S2 Search Results for Small Molecules Exhibiting Transcriptomic Signatures Similar to Linezolid in THP-1 Cells at 10 µM and 3.3 µM.

-10 µM linezolid

Perturbagen	Perturbation Type	Timepoint	z-score (sum)	p-value Bonferroni
linezolid	Chemical	24 h	34.49882	1.181e-319
amuvatinib	Chemical	24 h	17.08393	2.99E-08
bortezomib	Chemical	24 h	15.74528	3.424937e-318
moxifloxacin	Chemical	24 h	14.80332	3.08E-09

oglemilast	Chemical	24 h	13.85362	0.004416
arglabin	Chemical	24 h	13.57039	4.87E-05
EMD-1214063	Chemical	24 h	13.05975	0.004827
BRD-K82225283	Chemical	24 h	12.80584	8.69E-06
dasatinib	Chemical	24 h	12.40823	3.5E-05
HC-030031	Chemical	24 h	12.35414	6.11E-05

-3.3 µM linezolid

Perturbagen	Perturbation Type	Timepoint	z-score (sum)	p-value Bonferroni
linezolid	Chemical	24 h	34.49882	3.424937e-318
preladenant	Chemical	24 h	14.85278	3.08E-09
epinephrine	Chemical	24 h	14.53572	2.61E-05
progesterone	Chemical	24 h	14.36372	3.424937e-318
fosfomycin	Chemical	24 h	12.954	2.54E-05
donepezil	Chemical	24 h	12.86876	1.34E-07
dasatinib	Chemical	24 h	12.41334	8.31E-09
clinafloxacin	Chemical	24 h	12.21019	6.16E-10
TAK-285	Chemical	24 h	12.06863	0.002693
milacemide	Chemical	24 h	12.04866	3.22E-07
timofibrate	Chemical	24 h	12.04372	1.97E-08

Table S3 cryo-EM image processing statistics of TOP2 DNA binding domain.

**Sampling interval (Å/pix)**	0.9013
**N of movies (collected)**	750
**N of fractions**	60
**Exposure time (sec)**	6.09
**Dose rate (e/pix/sec)**	6

**Total dose (e/Å^2)**	60
**Defocus range (μm)**	−0.8 ~ −2.4 (0.1 and 0.2)
**Map refinement**	cryoSPARC v3.1.4
**Motion correction**	cryoSPARC v3.1.4
**CTF estimation**	Patch CTF estimation
**Particle picking**	cryoSPARC v3 (Blob picker)
**Particle box size (pixel × pixel)**	200 x 200
**N of particle images**	124,337
**3D refinement**	cryoSPARC v3.1.4 (NU-refinement)
**Symmetry imposed**	C1
**CTF refinement**	cryoSPARC v3.1.4
**GSFSC resolution (Å)**	4.21

Table S4 Zebrafish toxicity according to the drug concentration and time (hpf).

Linezolid-treated group													
n = 10	Mortality						Pericardial Edema (PE)						
Time (hpf)	72hpf		96hpf		120hpf		72hpf		96hpf		120hpf		
	Mean	SD	Mean	SD	Mean	SD	Mean	SD	Mean	SD	Mean	SD
0.1% DMSO	0	0.0	0	0.0	0	0.0	0	0.0	0	0.0	0	0.0
0.0005 mM	0	0.0	0	0.0	0	0.0	0	0.0	0	0.0	0	0.0
0.005 mM	0	0.0	0	0.0	0	0.0	0	0.0	1	1.0	0	0.0

0.025 mM	0	0.0	0	0.0	0	0.0	0	0.0	1	1.0	1	1.0
0.125 mM	0	0.0	0	0.0	3	5.0	1	0.0	3	4.2	1	0.0
0.25 mM	0	0.0	0	0.0	3	5.0	0	0.0	2	3.5	0	0.0
0.5 mM	0	0.0	0	0.0	1	1.0	1	0.0	3	4.6	3	0.5
n = 10	Yolk sac edema (YSE)						Bent tail (BT)					
Time (hpf)	72hpf		96hpf		120hpf		72hpf		96hpf		120hpf	
	Mean	SD	Mean	SD	Mean	SD	Mean	SD	Mean	SD	Mean	SD
0.1% DMSO	0	0.0	0	0.0	0	0.0	0	0.0	0	0.0	0	0.0
0.0005 mM	0	0.0	0	0.0	0	0.0	0	0.0	0	0.0	0	0.0
0.005 mM	0	0.0	0	0.0	0	0.0	0	0.0	0	0.0	0	0.0
0.025 mM	0	0.0	0	0.0	1	1.0	0	0.0	0	0.0	1	1.0
0.125 mM	0	0.0	0	0.0	0	0.0	0	0.0	0	0.0	0	0.0
0.25 mM	0	0.0	1	2.5	0	0.0	0	0.0	0	0.0	0	0.0
0.5 mM	0	0.0	0	0.5	0	0.5	0	0.0	0	0.0	0	0.5
PNU142586-treated group												
n = 10	Mortality						Pericardial Edema (PE)					
Time (hpf)	72hpf		96hpf		120hpf		72hpf		96hpf		120hpf	
	Mean	SD	Mean	SD	Mean	SD	Mean	SD	Mean	SD	Mean	SD
0.1% DMSO	0	0.0	0	0.0	0	0.0	0	0.0	0	0.0	0	0.0
0.0005 mM	0	0.0	0	0.0	0	0.0	0	0.0	0	0.0	0	0.0
0.005 mM	0	0.0	0	0.0	0	0.5	0	0.0	1	1.0	0	0.0
0.025 mM	0	0.0	0	0.0	0	0.0	0	0.0	0	0.0	0	0.0
0.125 mM	0	0.0	0	0.5	1	0.6	2	0.5	1	0.0	1	0.6
0.25 mM	0	0.0	0	0.0	1	1.0	2	0.0	1	0.8	1	0.5

0.5 mM	0	0.0	0	0.0	0	0.0	1	0.0	1	0.5	0	0.6
n = 10	Yolk sac edema (YSE)						Bent tail (BT)					
Time (hpf)	72hpf		96hpf		120hpf		72hpf		96hpf		120hpf	
	Mean	SD	Mean	SD	Mean	SD	Mean	SD	Mean	SD	Mean	SD
0.1% DMSO	0	0.0	0	0.0	0	0.0	0	0.0	0	0.0	0	0.0
0.0005 mM	0	0.0	0	0.0	0	0.0	0	0.0	0	0.0	0	0.0
0.005 mM	0	0.0	0	0.0	0	0.0	0	0.0	0	0.0	0	0.0
0.025 mM	0	0.0	0	0.0	0	1.0	0	0.0	0	0.0	0	0.0
0.125 mM	0	0.5	1	0.6	1	0.6	0	0.0	0	0.0	0	0.5
0.25 mM	0	0.0	0	0.5	0	0.5	0	0.5	0	0.5	1	0.6
0.5 mM	0	0.0	0	0.0	1	0.6	0	0.0	0	0.0	0	0.0

Table S5 Steady state concentration ranges of linezolid and its primary metabolites according to regimen

Regimen	Concentrations (mg/L)[a]			Resource
	**linezolid**	**PNU142586**	**PNU142300**	
600mg BID (twice daily)	10.8 (6.3 – 17.4)	11.4 (6.2 – 19.4)	4.4 (2.5 – 6.6)	E. Souza et al. *(33)*
600mg QD (once daily)	5.4 (3.15 – 8.7)	5.7 (3.1 – 9.7)	2.2 (1.25 – 3.3)	Derived from E. Souza et al. *(33)*[b]

[a]Data are presented as medians (Interquartile range)

[b]The data for 600 mg QD were derived from the results of 600 mg BID using the equation of $C_{ss,avg} = {}^{Dose}/_{CL} \times \tau$, where $C_{ss,avg}$ is average steady stat concentration, Dose is administered drug amount, CL is clearance, and τ is dosing frequency, based on the assumptions that the drug exhibits linear pharmacokinetics (allowing dose-proportionality to be applied) and that the concentration range is directly proportional to the average steady-state concentration.

Table S6 List of primers used in this study.

Gene	Primer sequence	Organism	Source	Storage
MTCO1	FP- CCACCTCTAGCCTAGCCGTTTA<br>RP- GGGTCATGATGGCAGGAGTAAT	Human	Macrogene	-20ºC
GADPH	FP- GAAGGTGAAGGTCGGAGTC<br>RP- GAAGATGGTGATGGGATTTC	Human	Macrogene	-20ºC
ef1α	FP- GGAGACTGGTGTCCTCAA<br>RP- GGTGCATCTCAACAGACTT	Zebrafish	Macrogene	-20ºC
mtco1	FP- ATATCGCCCTACCAATCGCA<br>RP- TGATCTCTTGGGCATGGGTT	Zebrafish	Macrogene	-20ºC

Data S1 Force field parameters of the analyzed compounds This file contains the force field parameters for linezolid, PNU142586, and PNU142300, including the corresponding .gro, .itp, and .prm files used in molecular dynamics simulations.
